# Mebendazole is unique among tubulin-active drugs in activating the MEK–ERK pathway

**DOI:** 10.1038/s41598-020-68986-0

**Published:** 2020-08-04

**Authors:** Claes R. Andersson, Tove Selvin, Kristin Blom, Jenny Rubin, Malin Berglund, Malin Jarvius, Lena Lenhammar, Vendela Parrow, Angelica Loskog, Mårten Fryknäs, Peter Nygren, Rolf Larsson

**Affiliations:** 1 Division of Cancer Pharmacology and Computational Medicine, Department of Medical Sciences, Uppsala University, 75185 Uppsala, Sweden; 2 Department of Immunology, Genetics and Pathology, Section of Oncology, Uppsala University, 75185 Uppsala, Sweden

**Keywords:** Pharmacology, Drug development

## Abstract

We recently showed that the anti-helminthic compound mebendazole (MBZ) has immunomodulating activity in monocyte/macrophage models and induces ERK signalling. In the present study we investigated whether MBZ induced ERK activation is shared by other tubulin binding agents (TBAs) and if it is observable also in other human cell types. Curated gene signatures for a panel of TBAs in the LINCS Connectivity Map (CMap) database showed a unique strong negative correlation of MBZ with MEK/ERK inhibitors indicating ERK activation also in non-haematological cell lines. L1000 gene expression signatures for MBZ treated THP-1 monocytes also connected negatively to MEK inhibitors. MEK/ERK phosphoprotein activity testing of a number of TBAs showed that only MBZ increased the activity in both THP-1 monocytes and PMA differentiated macrophages. Distal effects on ERK phosphorylation of the substrate P90RSK and release of IL1B followed the same pattern. The effect of MBZ on MEK/ERK phosphorylation was inhibited by RAF/MEK/ERK inhibitors in THP-1 models, CD3/IL2 stimulated PBMCs and a MAPK reporter HEK-293 cell line. MBZ was also shown to increase ERK activity in CD4+ T-cells from lupus patients with known defective ERK signalling. Given these mechanistic features MBZ is suggested suitable for treatment of diseases characterized by defective ERK signalling, notably difficult to treat autoimmune diseases.

## Introduction

Mebendazole (MBZ) is an anti-parasitic drug that has gained considerable interest as a potential anticancer agent. MBZ is generally perceived as a tubulin polymerase inhibitor^1,2^ but several other mechanisms have been suggested to explain the anticancer activity observed in vitro and in vivo. Mechanisms suggested include angiogenesis inhibition^3,4^, apoptosis induction^5,6^ and inhibition of the Hedgehog signalling pathway^7^. MBZ has also been shown to potently bind to several protein kinases involved in oncogenic signalling^8^. Anticancer activity has also occasionally been observed in patients^9,10^.

Recently, we demonstrated that MBZ induces a pro-inflammatory tumour-suppressive M1 phenotype in THP-1 monocytes and macrophages which could potentially explain tumour cell killing^11^. It was also noted that MBZ induced activation of ERK potentially contributes to the immune effects observed.

ERK is part of a pathway which comprises proteins referred to as mitogen-activated protein kinases (MAPKs)^12^. The MAPKs can be categorised into three major protein families: extracellular signal-regulated kinases (ERK), stress-activated protein kinases/c-Jun N-terminal kinase (SAPK/JNK) and the p38 proteins^12^. Whereas SAPK/JNK is associated with stress response, apoptosis and inflammation, increased ERK is generally suggested to mediate cell survival, activation and differentiation^13^. Proximal proteins involved in the ERK pathway include Ras, Raf and MEK^12^.

In the present study we investigated whether MBZ induced ERK activation is shared by other tubulin binding agents (TBAs) and if the ERK activation is observable also in other human cell types.

## Methods

### Cell culture

Monocytoid THP-1 cells were obtained from ATCC (Manassas, VA, USA) and were cultured in RPMI-1640 medium, supplemented with 10% heat-inactivated foetal bovine serum (HIFBS), 2 mM l-glutamine, 100 U/100 µg/ml penicillin/streptomycin and 0.05 mM 2-mercaptoethanol (all from Sigma, St Louis, MO, USA). For differentiation and polarisation of THP-1 cells to macrophages, 25 ng/mL PMA, 20 ng/mL IFNγ, 100 ng/mL LPS, 20 ng/mL IL4 and 20 ng/mL IL13 (final concentrations) were used according to an established protocol with minor modifications^14^. The SRE Reporter HEK293 cell line was obtained from BPS Bioscience (San Diego, CA, USA) and used for monitoring the activity of the MAPK/ERK signalling pathway. The SRE Reporter HEK293 cell line contains a firefly luciferase gene under the control of Serum Response Element (SRE) responsive elements stably integrated into HEK293 cells, resulting in an ERK pathway-responsive reporter cell line. All cell lines were cultured at 37 °C in a humidified atmosphere containing 5% CO2. CD4+ T-cells from patients with SLE were purchased from Astarte Biologics, LLC (Bothell, WA, USA) and handled according to the manufacturer’s recommendations.

### Materials

Mebendazole (MBZ), albendazole (ABZ), fenbendazole (FBZ), lipopolysaccharide (LPS), interferon gamma (IFNγ), IL4, IL13 and phorbol myristate acetate (PMA) were purchased from Sigma (St Louis, MO, USA). The ERK/MEK/Raf inhibitors used were purchased from Selleckchem (Houston, TX, USA). The compounds were kept as 10 mM stock solutions in dimethyl sulphoxide (DMSO, Sigma) or sterile water, and further diluted with culture medium (Sigma or ATCC) as needed.

### Bioinformatic analysis using the LINCS L1000 platform

The drug-induced gene expression perturbations of MBZ were studied using the public LINCS Connectivity Map (CMap) resource (clue.io, formerly www.lincscloud.org)^15,16^ that contains a collection of hundreds-of-thousands of L1000 gene-expression profiles from cells grown in monolayer exposed to large numbers of small-molecule and genetic perturbagens. Since MBZ is present in the database, the gene expression profile can be compared with those of other drugs and perturbagens. Score and ranking were retrieved from the LINCS CMap database using default settings (best 4 cell lines). THP-1 gene expression data obtained using the L1000 assay (see below) were uploaded and analysed in a similar manner.

For the L1000 assay, drugs and tool compounds were transferred to monolayer plates using Echo Liquid Handler 550 (Labcyte Inc, Sunnyvale, CA). Following 1 h or 6 h incubation, the cell-culture medium was aspirated and cell lysis buffer (Genometry, Inc., Boston, MA, USA) added. After 30 min incubation at room temperature, cell samples were mixed. Homogeneous lysates were transferred to 384-well Nunc plates (Thermo Fisher Scientific, Waltham, MA, USA) and frozen at − 70 °C. Lysates were processed, and the resulting gene expression data subjected to a panel of quality control tests, performed by Genometry in their facility (www.genometry.com). The L1000 expression profiles delivered were scaled, normalized and log transformed, and denominated in the Affymetrix HG-U133A feature space.

### Measurement of cytokines and phosphoproteins

The levels of cytokines and phosphoproteins were measured using the Luminex MAGPIX system and commercially available kits for various analytes (Bio-Rad, Hercules, CA, USA) and were performed as previously described^11^ according to the manufacturer’s instructions. The target of interest is bound to magnetic beads via antibodies, and detected using biotinylated antibodies with a fluorescent reporter. Briefly, in the cytokine assay the supernatant samples were incubated firstly with beads, secondly with detection antibody and finally with streptavidin-PE. Fluorescence was measured using the MAGPIX instrument (Bio-Rad) and concentration levels were determined using a fitted standard curve. For phosphoprotein assays, protein concentrations in the lysates were measured using a Micro BCA method (Thermo Fisher Scientific) in order to ensure equal amounts of samples in the assay before measuring using the same protocol as for the cytokine assay.

### Measurement of tubulin polymerization

Tubulin polymerization from purified tubulin monomers was measured as increased fluorescence because of the incorporation of a fluorescent reporter into growing microtubules as previously described^11^. Reagents necessary for performing the assay were provided in the kit BK011 from Cytoskeleton (Denver, Colorado, USA) and fluorescence was measured at 1 min intervals for 60 min using a FLUOstar Omega (BMG Labtech GmbH, Offenburg, Germany).

### Measurement of cell cycle

Cell cycle was assayed in THP-1 cells. These cells were plated in RPMI-1640 medium with 10% heat inactivated foetal bovine serum, 2 mM glutamine, 100 µg/ml streptomycin and 100 units/ml penicillin at 1 × 10^6^ cells/well in 24-well plates. Test compounds were added to cells at 10 times the final concentration in medium and incubated at 37 °C 5% CO2 overnight. On the day of assay cells were permeabilized, the DNA stained with fluorescent dye (DAPI) and analysed according to the manufacturer’s instructions with “2-step Cell Cycle Assay” on a NucleoCounter NC-3000 (ChemoMetec A/S, Allerod, Denmark). Cellular fluorescence was quantified, and DNA content histograms were displayed. The phases of the cell cycle were gated and data collected.

## Results

**Figure 1:**
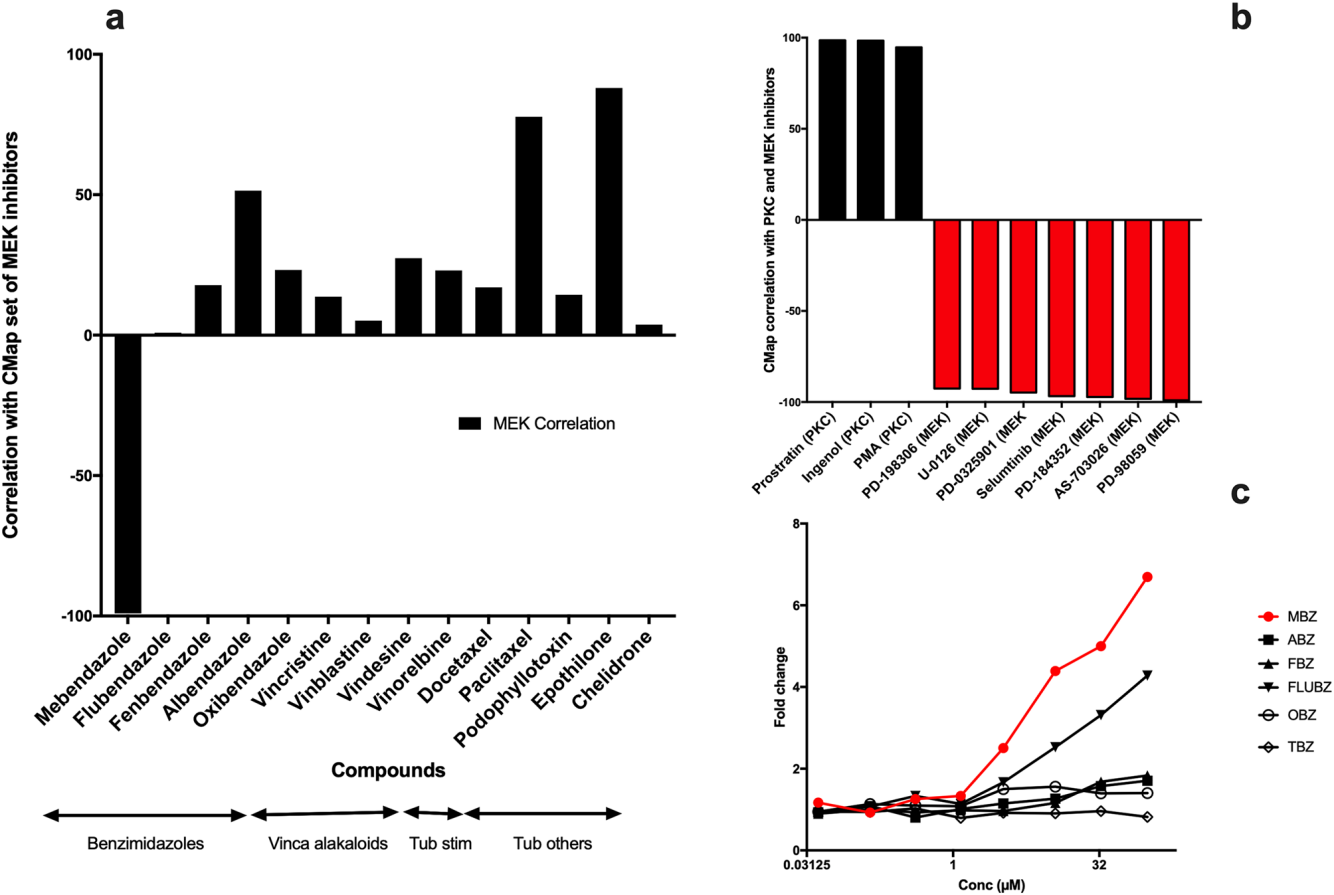
Gene expression analysis of MBZ. Correlation of MBZ and a panel of TBAs with MEK inhibitors in the CMap database (**a**). Correlation of uploaded L1000 data (see material and methods) in response to 10 µM MBZ with PKC and MEK inhibitors (**b**). Effect of MBZ and other benzimidazoles on expression of a marker of ERK activity, DUSP6, using the L1000 platform (**c**).

First, we performed a bioinformatic analysis to investigate whether the activation of ERK is unique to MBZ among tubulin binding agents (TBAs). We took advantage of the LINCS connectivity map database using the gene expression signatures for MBZ and a panel of well-known and clinically used TBAs as queries. The resulting drug-specific scores are based on the aggregated response from a panel of 6 non-haematological cell lines. MBZ showed strong negative correlations (− 99.7) to a predefined set of 8 MEK/ERK inhibitors in CMap which was not the case for the other TBAs showing correlations ranging between (+ 4 and + 77, Fig. 1a). These results indicate that MBZ but not the remaining TBAs strongly activates the MEK/ERK pathway.

Uploading our own MBZ induced gene expression L1000 signature from the THP-1 cell line also showed strong negative correlations with the MEK/ERK inhibitors (Fig. 1b). The highest positive correlations were observed for a set of PKC activators (Fig. 1b) which are known to induce ERK dependent THP-1 adhesion and differentiation^17^. The L1000 gene expression of DUSP6, a marker gene for ERK activation, dose dependently increased only in response to MBZ and to some extent flubendazole but no other benzimidazoles. (Fig. 1c). Finally, using the Reactome pathway analysis tool queried with the MBZ gene L1000 signature six of 25 top pathways enriched involved MAPK signalling (Supplementary Data).

**Figure 2:**
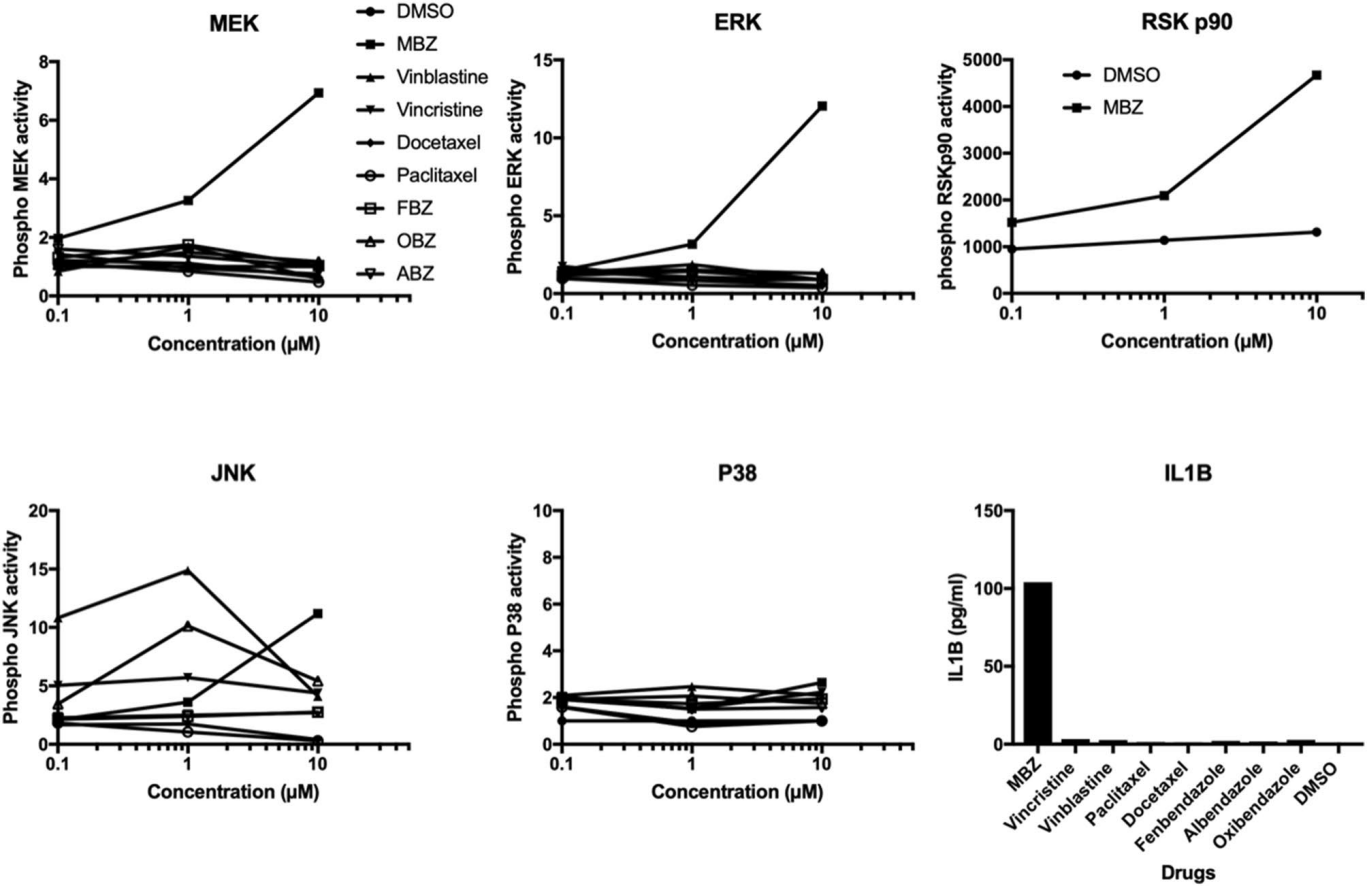
Effect of MBZ and a panel of TBAs on MAPK phosphoprotein activity in naive THP-1 monocytes. In the last panel the effect of 10 µM MBZ and TBAs on IL1B release is shown. The experiments were repeated at least two times with similar results. *MBZ* mebendazole, *FBZ* fenbendazole, *OBZ* oxibendazole, *ABZ* albendazole.

Next, we employed a multiplex Luminex based assay to directly measure the effects of MBZ and the TBAs on MAPK phosphoprotein activity at 0.1, 1 and 10 µM using naive THP-1 cells as the model (Fig. 2**)**. Again only MBZ increased MEK and ERK signalling with little or no effect observed for the other TBAs. MBZ also increased the activity of the ERK substrate P90RSK. Vinblastine and vincristine, on the other hand, increased primarily SAPK/JNK activity which is consistent with the literature^13^, whereas this was observed for MBZ only at the highest concentration (10 µM, Fig. 2). None of the TBAs, including MBZ, affected p38 phosphorylation.

**Figure 3:**
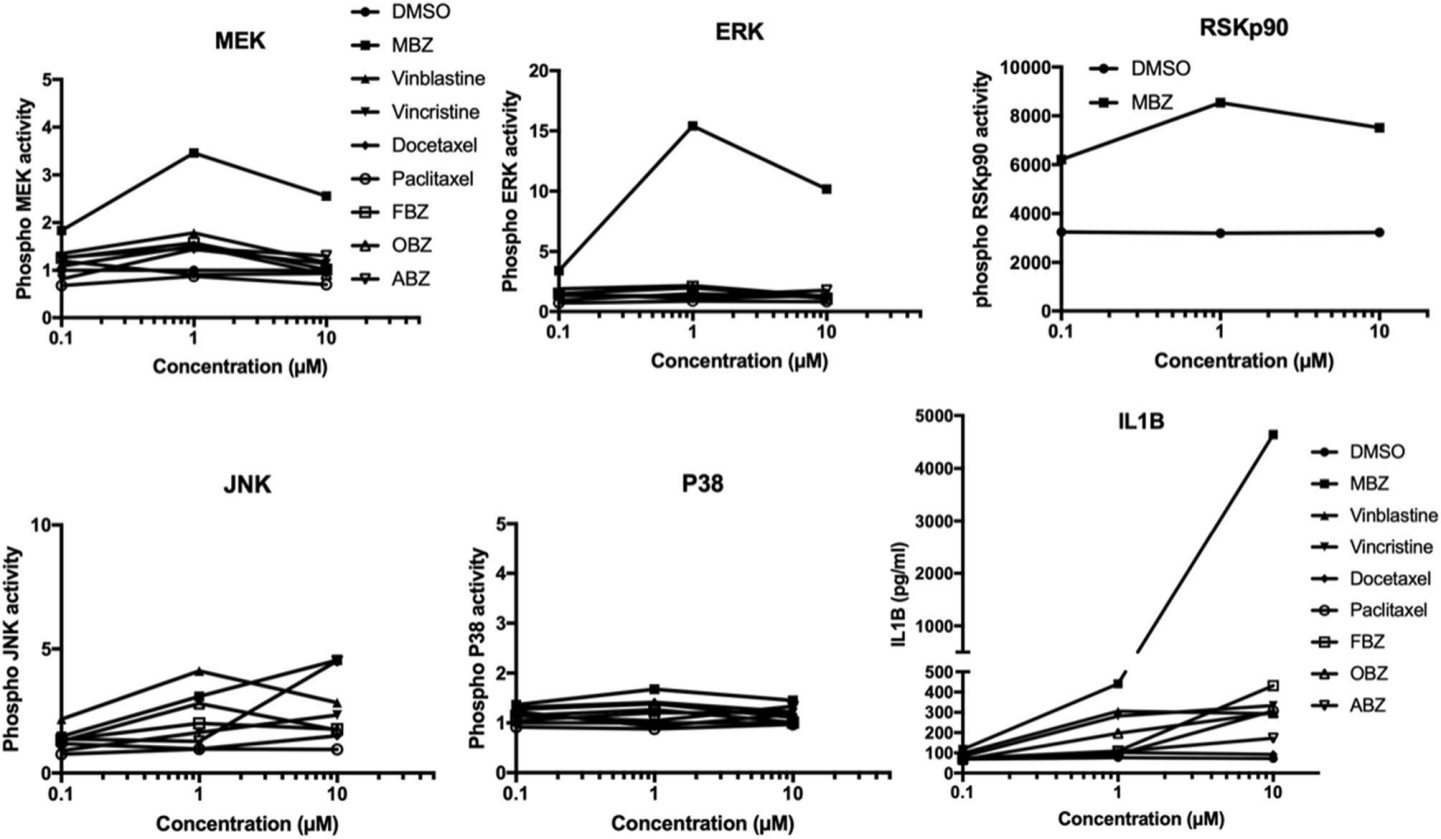
Effect of MBZ and a panel of TBAs on MAPK phosphoprotein activity and IL1B release in PMA differentiated THP-1 macrophages. The experiments were repeated at least two times with similar results. *MBZ* mebendazole, *FBZ* fenbendazole, *OBZ* oxibendazole, *ABZ* albendazole.

When distal effects on cytokine release was measured only MBZ showed a significant effect on IL1B secretion. Very similar results were obtained in PMA differentiated THP-1 macrophages (Fig. 3). Interestingly in this model the effect of MBZ appeared potentiated causing ERK activation at lower concentrations. The above results in both THP-models were observed at concentrations of TBAs that affected tubulin polymerisation (Supplementary Fig. S1) and induced G2/M arrest (Supplementary Fig. S2).

**Figure 4:**
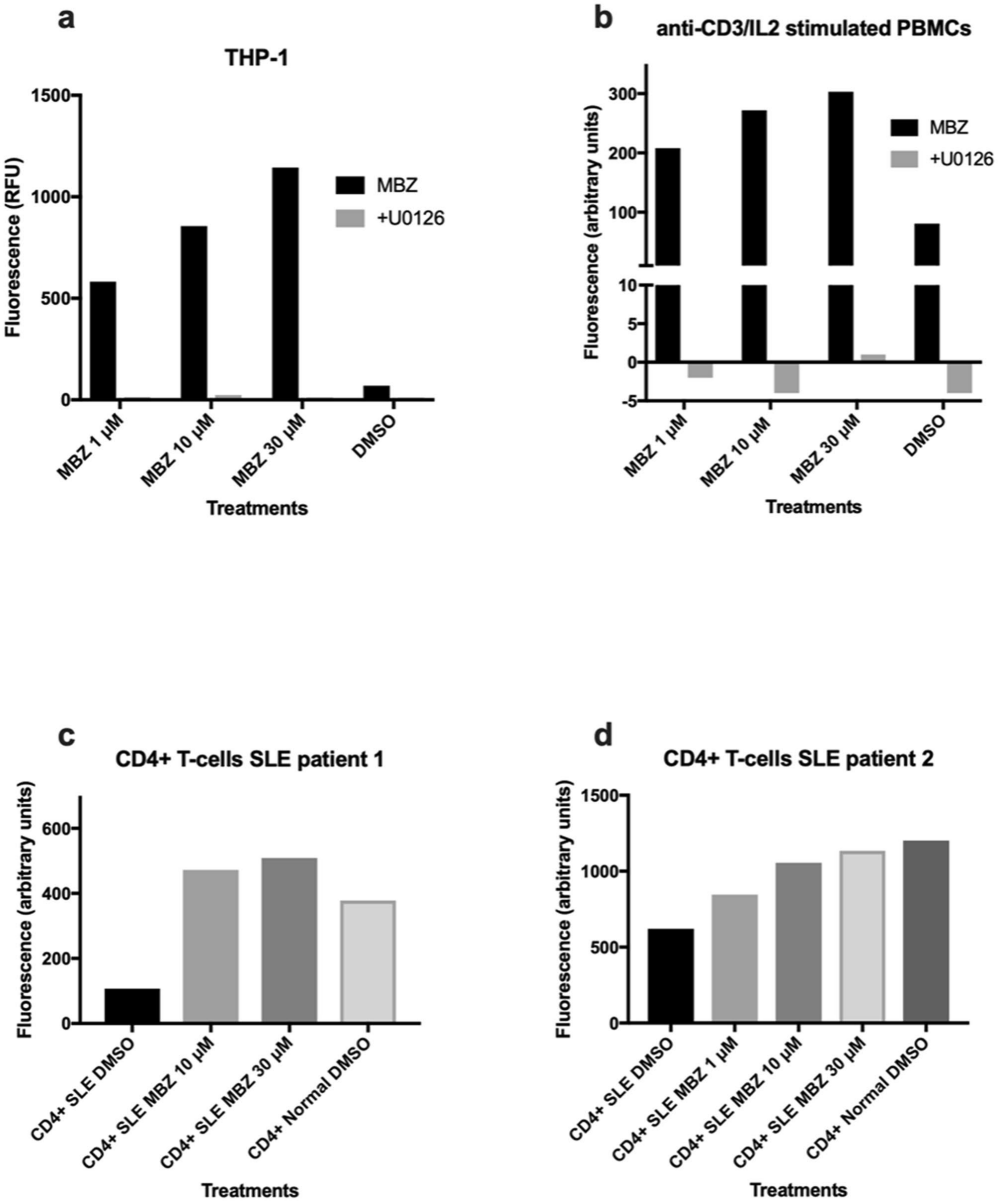
Effect of MBZ with or without MEK inhibitor U0126 on ERK activity in THP-1 monocytes (**a**), PBMC activated by antiCD3/IL2 (**b**) and CD4+ T-cells from patients with SLE (**c**,**d**).

**Figure 5:**
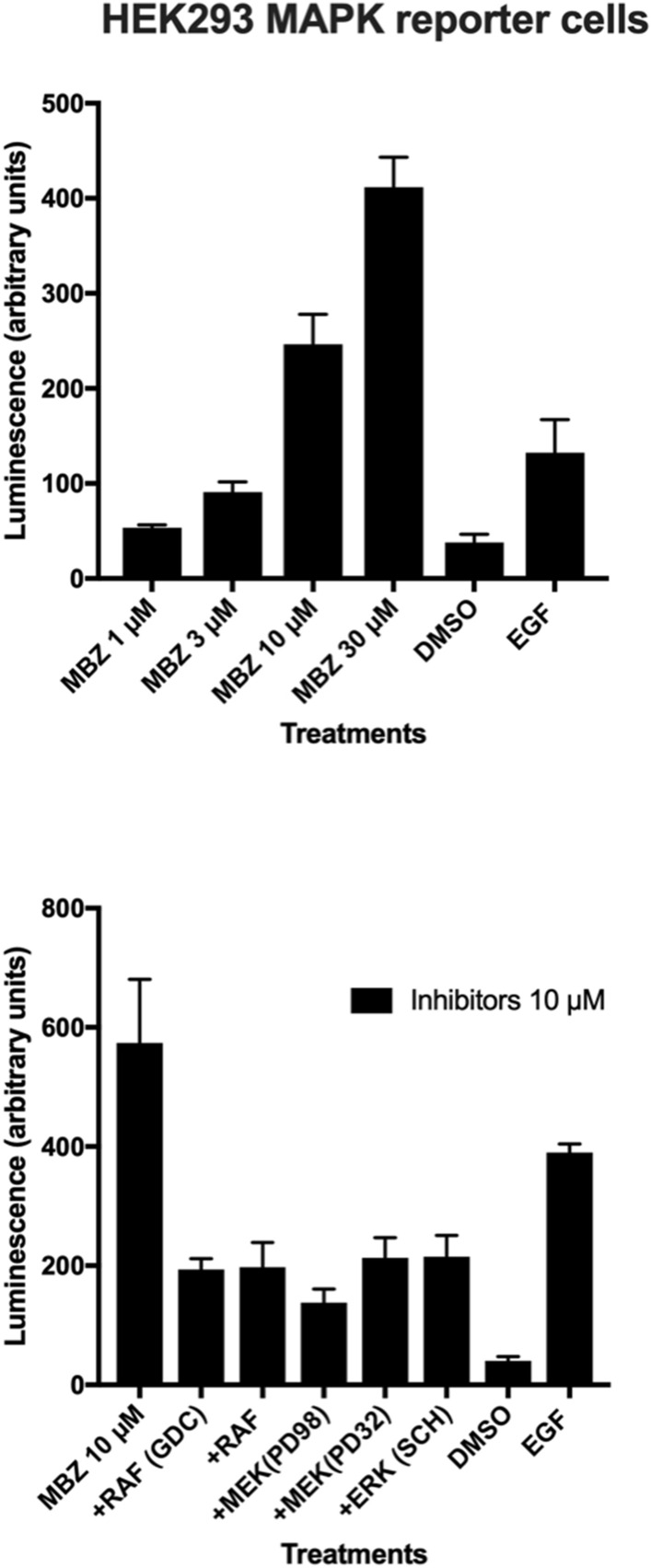
Effect of MBZ and positive control EGF on ERK activity in MAPK reporter HEK293 cell line (**a**). In (**b**) the effect of MBZ with and without different ERK/MEK/Raf inhibitors is shown. The results are expressed as mean values + / − SEM for three independent experiments.

The kinetics of MBZ induced ERK signalling appeared to be biphasic with one peak observed after 1 h followed by a decline and then increasing again at 6–24 h (Supplementary Fig. S3). Interestingly, in the THP-1 model MBZ differed from the classical M1 stimuli LPS/IFN, the latter increasing p38 signalling including downstream Hsp27 activity whereas MBZ primarily affected MEK/ERK phosphorylation (Supplementary Fig. S3). Increased ERK activity upon MBZ treatment was observed in THP-1 cells as well as in human PBMCs stimulated with anti-CD3 antibody and IL-2. The effect was completely abrogated by the MEK inhibitor U0126 (10 µM). Furthermore, MBZ increased ERK activity in CD4+ T-cells from SLE patients with known defective ERK signalling (Fig. 4). Finally, we employed a MAPK reporter HEK293 cell line and demonstrated that MBZ readily increased reporter activity even stronger than the positive control EGF (Fig. 5). Moreover, this activity was inhibited by selective RAF, MEK and ERK inhibitors (Fig. 5).

## Discussion

In the present study we demonstrated that MBZ uniquely induce ERK activation in several cell types including those of non-haematological origin. Bioinformatic analysis has been used to identify pathogenetic mechanisms in diseases of different origins including immunoinflammatory diseases and cancer and helps to predict novel therapeutic targets as well as elucidate mode and mechanism of action^18,19^. Here we used a database of transcription drug response signatures, CMap, to compare MBZ to other TBAs. The striking negative correlation to MEK inhibitors in CMap is based on solid tumour cell lines only and was not observed for other compounds, including TBAs, in the database.

In the literature TBAs are long known to activate MAPK signalling but this effect has mostly been associated with JNK activation^13^ and TBAs have been reported to inhibit ERK in several cell systems^20–22^. This was confirmed in the present study in which only MBZ but no other TBAs, including other benzimidazoles, activated ERK to any significant extent. However, some of them indeed activated SAPK/JNK in accordance with the literature^13^. In the present study the tested concentrations of MBZ were relatively high (often 1–30 µM). However in most of the experimental models tested a clear effect on MEK/ERK activation was observed at 1 µM which is a plasma concentration achievable in the clinical setting^23^.

**Figure 6:**
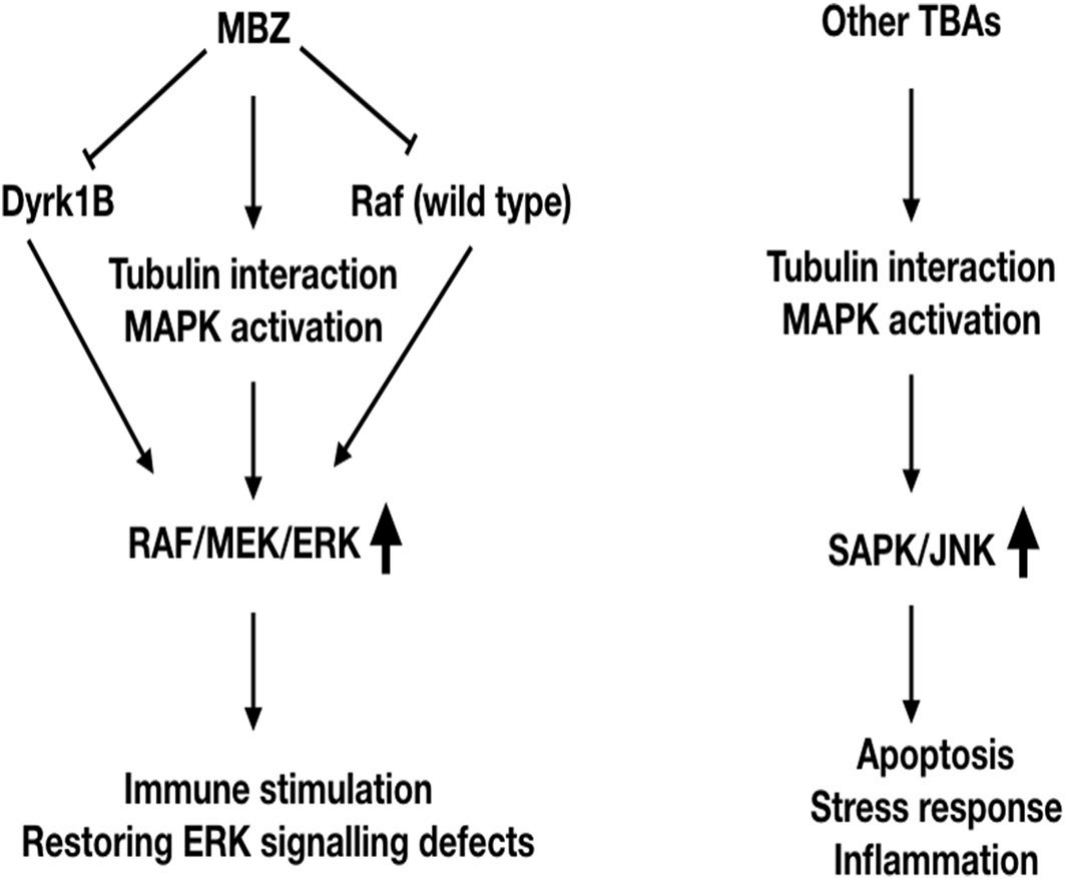
Hypothesis explaining the difference between MBZ and other TBAs for MAPK activation. TBAs are known to activate MAPK signalling, however, mainly through SAP/JNK. MBZ but no other benzimidazoles activate ERK. Additionally, MBZ inhibits DYRK1b which can lead to ERK activation, as well as potently inhibits BRAF which in cells with wild type RAF leads to paradoxical ERK activation.

The reason for the MBZ selective ERK activation is still not clear but is apparently not only due to tubulin depolymerization per se. One potential explanation could involve specific interactions with the tubulin structure. MBZ is known to bind to the colchicine binding site of tubulin but this is also the case for other benzimidazoles^24^, thus providing no simple explanation for the MBZ induced selective ERK activation. However, MBZ has been demonstrated to inhibit DYRK1b at low nM concentrations which can lead to ERK activation^25,26^. Moreover, MBZ also potently inhibits BRAF^8^ which in cells with wild type RAF leads to paradoxical ERK activation^27,28^. Thus, RAF inhibitors selectively block ERK signalling in BRAF-mutant tumours but have the opposite effect in BRAF wild-type cells such as T-cells, where they cause hyper-activation of ERK signalling^28^. These additional pharmacodynamic effects may consequently contribute to the observed MBZ induced ERK activation. The potential mechanisms behind MBZ-induced ERK activation are depicted in Fig. 6.

In cancer MAPK/ERK activation has mostly been associated with tumour growth promotion resulting from overactivity in RAS and RAF mutated signalling pathways^12^. However, in certain cell types and contexts ERK activation can, on the contrary, be a requirement for inducing cell death^29,30^. With respect to immune stimulation ERK is necessary for activating immune cells, both T-cells^31^ and macrophages^32^, and inducing immune cell mediated tumour cell killing^33^. For tumours with RAS or BRAF mutations with already maximal intrinsic ERK signalling, ERK mediated immune cell activation may shift the balance towards tumour suppression. However, for some tumour types growth promotion cannot be excluded.

In the immune-oncology setting studies have shown that engagement of PD-1 by PD-L1 inhibit the MEK/ERK MAPK pathway leading to suppression of T-cell activation and proliferation^34,35^. The effect of PD-1 on MEK/ERK and MAP kinases was found to be selective because PD-1 ligation could not inhibit the activation of JNK and p38 MAP kinases^34^. Also in macrophages signals from inhibitory receptors can downregulate the MEK/ERK pathway leading to inhibition of macrophage activation and function^36^. One example is the interaction of CD200 ligand (expressed on tumour cells) with CD200 receptors (CD200R) on myeloid cells which negatively regulates the activation of these cells secondary to MEK/ERK inhibition. CD200R expression is also associated with M2 polarisation of macrophages^37^. We speculate that MBZ may counteract these immune suppressive effects by restoring ERK activity. We have also previously shown that MBZ induces an M1 phenotype in human macrophage models and potentiates the anti-cancer activity of CD3/IL-2 activated peripheral blood mononuclear cells (PBMCs), the effect being attenuated by removal of CD14+ myeloid cells^38^.

However, ERK is not always a signal for immune stimulation and inflammation but can in certain cells and situations exert negative feedback on p38/JNK driven inflammation^39,40^. One explanation for this reciprocal feedback activity has been attributed to MAPK crosstalk. Both increased p38 and JNK activity can inhibit ERK through activation of PP2 and AP-1 transcription, respectively^41^. ERK activation, on the other hand, increases MKP1 protein stability and availability which in turn causes preferentially de-phosphorylation and inactivation of p38 and JNK^39,40^. The resulting decrease in p38 and JNK activity downregulates the pro-inflammatory response. ERK has also been shown to inhibit NF^42,43^. These potential mechanisms are schematically depicted in Supplementary Fig. S4.

Defective ERK signalling has been implicated in some autoimmune diseases including sarcoidosis^44^ and SLE^45–47^, with strongest evidence presented for the latter disease. For example, inhibition of MEK/ERK signalling by agents such as hydralazine and other MEK inhibitors can induce lupus and lupus-like autoimmune diseases^45,47^. Furthermore, decreased ERK activity in CD4+ T-cells obtained from SLE patients has been suggested to be a key factor in development of SLE by causing DNA hypomethylation, and consequent aberrant gene expression and disease manifestation^45–47^. The present study supports the potential utility of MBZ for increasing and normalising ERK activity in CD4+ cells from patients with SLE. Another postulated pathogenetic mechanism involves type I interferons released from plasmacytoid dendritic cells (PDC) believed to be a key initiating event in the development of autoimmune disease including SLE^48^. Indeed also in this case activation of the MEK/ERK pathway inhibits type I interferon release from PDCs^49^.

Given the mechanistic features suggested here, MBZ may be therapeutically useful also in autoimmune disease driven by Type I-interferons and/or p38 which may be ameliorated by ERK activation, such as systemic sclerosis, myositis, multiple sclerosis, Sjögren’s disease, rheumatoid arthritis and psoriasis^48,50–52^. However, it should be noted that for some of these diseases such as rheumatoid arthritis and multiple sclerosis there are conflicting reports demonstrating that inhibition of the MEK/ERK could ameliorate disease manifestation^53,54^ suggesting a more complex and different pathophysiology. For example, the pleiotropic proinflammatory cytokine macrophage migration inhibitory factor (MIF) which activates the MEK/ERK pathway is upregulated and centrally implicated in the pathogenesis of both multiple sclerosis and rheumatoid arthritis^55–57^. On the other hand, glatiramer acetate, an immunomodulating drug used for treatment of multiple sclerosis has been shown to activate MEK/ERK which potentially underlies the ability of glatiramer acetate to induce anti-inflammatory IL-1 receptor antagonist production^58^. Thus, the potential role of MEK/ERK signalling in autoimmune diseases indirectly suggested based on the association with type-I interferon and/or p38 signalling is clearly hypothetical and needs to be confirmed. Notably, only in SLE is there ample experimental evidence for defective ERK signalling being directly linked to disease pathophysiology^45–47,49^. Further supporting SLE as a potential target diagnosis for MBZ we have observed that MBZ significantly inhibits anti-dsDNA production and formation of immune complex deposits in the kidney without noticeable toxicity in the spontaneous NZB/W F1 mouse model of SLE (unpublished preliminary results).

Finally, MBZ has shown clinical activity in the diabetes setting^59^. In this clinical study MBZ increased insulin secretion and decreased plasma glucose levels in Type 1 and Type 2 diabetic patients^59^. Follow up experiments on isolated islets demonstrated that MBZ potentiated insulin release in the presence of stimulatory concentrations of glucose^60^. In the context of ERK activation recent studies may shed light on these observations demonstrating that ERK activation is associated with potentiation of glucose induced insulin secretion^61,62^.

In conclusion, in the present study we demonstrate that MBZ induced ERK activation is not mimicked by other tubulin active agents and occurs in both haematological and non-haematological cell types. This unique feature of MBZ is suggested to be utilised to treat conditions with defective ERK signalling.

## Supplementary information

Supplementary Data.

Supplementary Figures.

## Data Availability

The datasets generated during and/or analysed during the current study are available from the corresponding authors on reasonable request.
