# Supplementary material for: Mebendazole is unique among tubulin-active drugs in activating the MEK–ERK pathway
Source: Sci Rep. 2020 Aug 4;10:13124. doi: 10.1038/s41598-020-68986-0 (PMC7403428; doi:10.1038/s41598-020-68986-0)
Supplement: Supplementary file 1 — Supplementary Data. [file 41598_2020_68986_MOESM1_ESM.pdf]

reactome

Pathway Analysis Report

Gene names MBZ 30

This report contains the pathway analysis results for the submitted sample 'Gene names MBZ 30'. Analysis was performed against Reactome version 65 on 17/07/2018 using any resource identifiers for the mapping. The web link to these results is:

https://reactome.org/PathwayBrowser/#/ANALYSIS=MjAxODA3MTcwNjU1MTFfMTUwNw%3D%3D

Please keep in mind that analysis results are temporarily stored on our server. The storage period depends on usage of the service but is at least 7 days. As a result, please note that this URL is only valid for a limited time period and it might have expired.

Table of Contents

1. Introduction
2. Properties
3. Genome-wide overview
4. Most significant pathways
5. Pathway details
6. Identifiers found
7. Identifiers not found

1. Introduction

Reactome is a curated database of pathways and reactions in human biology. Reactions can be considered as pathway 'steps'. Reactome defines a 'reaction' as any event in biology that changes the state of a biological molecule. Binding, activation, translocation, degradation and classical biochemical events involving a catalyst are all reactions. Information in the database is authored by expert biologists, entered and maintained by Reactome’s team of curators and editorial staff. Reactome content frequently cross-references other resources e.g. NCBI, Ensembl, UniProt, KEGG (Gene and Compound), ChEBI, PubMed and GO. Orthologous reactions inferred from annotation for Homo sapiens are available for 17 non-human species including mouse, rat, chicken, puffer fish, worm, fly, yeast, rice, and Arabidopsis. Pathways are represented by simple diagrams following an SBGN-like format.

Reactome's annotated data describe reactions possible if all annotated proteins and small molecules were present and active simultaneously in a cell. By overlaying an experimental dataset on these annotations, a user can perform a pathway over-representation analysis. By overlaying quantitative expression data or time series, a user can visualize the extent of change in affected pathways and its progression. A binomial test is used to calculate the probability shown for each result, and the p-values are corrected for the multiple testing (Benjamini–Hochberg procedure) that arises from evaluating the submitted list of identifiers against every pathway.

To learn more about our Pathway Analysis, please have a look at our relevant publications:

Fabregat A, Sidiropoulos K, Garapati P, Gillespie M, Hausmann K, Haw R, … D’Eustachio P (2016). The reactome pathway knowledgebase. Nucleic Acids Research, 44(D1), D481–D487. https://doi.org/10.1093/nar/gkv1351.

Fabregat A, Sidiropoulos K, Viteri G, Forner O, Marin-Garcia P, Arnau V, … Hermjakob H (2017). Reactome pathway analysis: a high-performance in-memory approach. BMC Bioinformatics, 18.

2. Properties

- This is an **overrepresentation** analysis: A statistical (hypergeometric distribution) test that determines whether certain Reactome pathways are over-represented (enriched) in the submitted data. It answers the question 'Does my list contain more proteins for pathway X than would be expected by chance?' This test produces a probability score, which is corrected for false discovery rate using the Benjamani-Hochberg method.
- 39 out of 47 identifiers in the sample were found in Reactome, where 354 pathways were hit by at least one of them.
- All non-human identifiers have been converted to their human equivalent.
- This report is filtered to show only results and pathway diagrams for Homo sapiens.
- The unique ID for this analysis (token) is MjAxODA3MTcwNjU1MTFfMTUwNw%3D%3D. This ID is valid for at least 7 days in Reactome's server. Use it to access Reactome services with your data.

3. Genome-wide overview

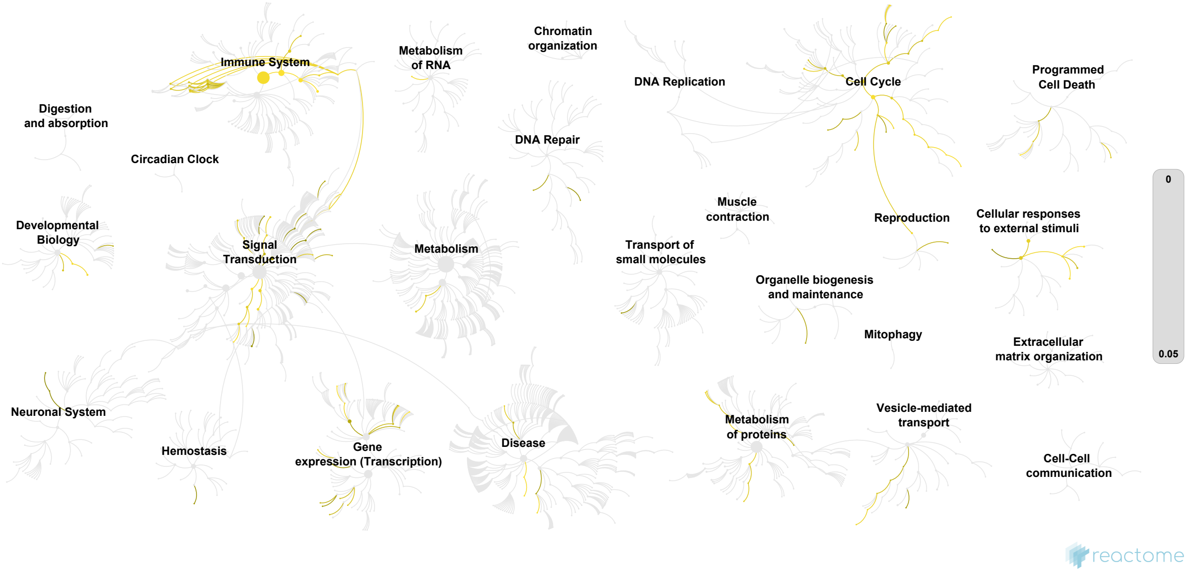

This figure shows a genome-wide overview of the results of your pathway analysis. Reactome pathways are arranged in a hierarchy. The center of each of the circular "bursts" is the root of one top-level pathway, for example "DNA Repair". Each step away from the center represents the next level lower in the pathway hierarchy. The color code denotes over-representation of that pathway in your input dataset. Light grey signifies pathways which are not significantly over-represented.

4. Top 25 pathways

Pathway name	Entities				Reactions	
	found	ratio	p-value	FDR*	found	ratio
Cytokine Signaling in Immune system	20 / 1,051	0.076	1.17e-07	4.89e-05	14 / 624	0.054
Signaling by Interleukins	14 / 640	0.046	2.77e-06	4.32e-04	10 / 491	0.042
Transcriptional activation of p53 responsive genes	3 / 6	4.33e-04	4.15e-06	4.32e-04	4 / 5	4.29e-04
Transcriptional activation of cell cycle inhibitor p21	3 / 6	4.33e-04	4.15e-06	4.32e-04	4 / 5	4.29e-04
Interleukin-4 and Interleukin-13 signaling	8 / 211	0.015	1.05e-05	8.72e-04	3 / 46	0.004
RAF-independent MAPK1/3 activation	4 / 28	0.002	1.29e-05	8.92e-04	2 / 12	0.001
Negative regulation of MAPK pathway	4 / 45	0.003	8.10e-05	0.005	2 / 12	0.001
TP53 Regulates Transcription of Genes Involved in G1 Cell Cycle Arrest	3 / 20	0.001	1.46e-04	0.008	6 / 17	0.001
Activation of NOXA and translocation to mitochondria	2 / 6	4.33e-04	4.31e-04	0.013	5 / 5	4.29e-04
TFAP2 (AP-2) family regulates transcription of cell cycle factors	2 / 6	4.33e-04	4.31e-04	0.013	3 / 4	3.44e-04
DNA Damage/Telomere Stress Induced Senescence	4 / 71	0.005	4.55e-04	0.013	7 / 18	0.002
Neurodegenerative Diseases	3 / 31	0.002	5.24e-04	0.013	8 / 22	0.002
Deregulated CDK5 triggers multiple neurodegenerative pathways in Alzheimer's disease models	3 / 31	0.002	5.24e-04	0.013	8 / 22	0.002
MAPK targets/ Nuclear events mediated by MAP kinases	3 / 35	0.003	7.43e-04	0.013	4 / 16	0.001
RUNX3 regulates CDKN1A transcription	2 / 8	5.77e-04	7.61e-04	0.013	3 / 6	5.15e-04
Senescence-Associated Secretory Phenotype (SASP)	4 / 89	0.006	0.001	0.017	8 / 22	0.002
Uptake and function of diphtheria toxin	2 / 10	7.21e-04	0.001	0.019	4 / 5	4.29e-04
MAPK family signaling cascades	7 / 328	0.024	0.001	0.019	9 / 86	0.007
Clearance of Nuclear Envelope Membranes from Chromatin	2 / 12	8.65e-04	0.002	0.024	1 / 1	8.59e-05
Cell Cycle	10 / 681	0.049	0.002	0.025	61 / 423	0.036
Insulin-like Growth Factor-2 mRNA Binding Proteins (IGF2BPs/IMPs/VICKZs) bind RNA	2 / 13	9.37e-04	0.002	0.025	3 / 3	2.58e-04

Pathway name	Entities				Reactions	
	found	ratio	p-value	FDR*	found	ratio
ERKs are inactivated	2 / 15	0.001	0.003	0.025	1 / 2	1.72e-04
Activation of HOX genes during differentiation	4 / 116	0.008	0.003	0.025	31 / 43	0.004
Activation of anterior HOX genes in hindbrain development during early embryogenesis	4 / 116	0.008	0.003	0.025	31 / 43	0.004
Nuclear Envelope Reassembly	2 / 16	0.001	0.003	0.027	1 / 3	2.58e-04

* False Discovery Rate

5. Pathway details

1. Cytokine Signaling in Immune system (R-HSA-1280215)

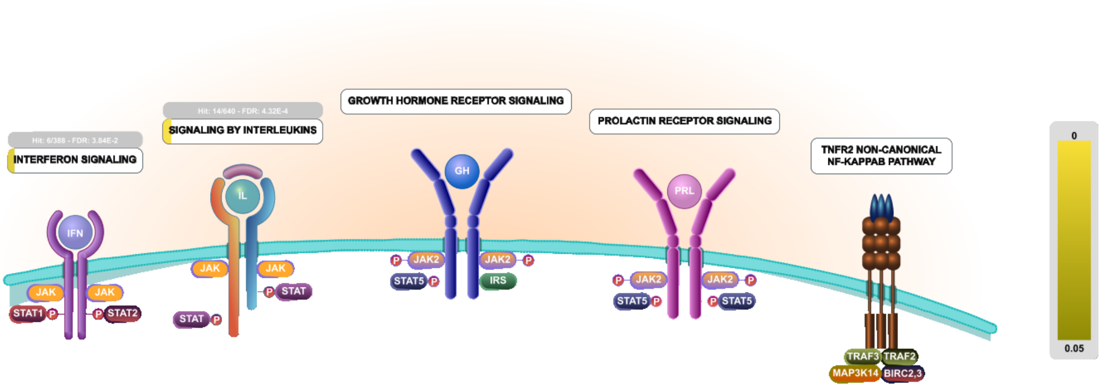

Cytokines are small proteins that regulate and mediate immunity, inflammation, and hematopoiesis. They are secreted in response to immune stimuli, and usually act briefly, locally, at very low concentrations. Cytokines bind to specific membrane receptors, which then signal the cell via second messengers, to regulate cellular activity.

References

Oppenheim J & Feldmann M (2002). *Cytokines and the immune system, Cytokine Reference* .

IMMPORT:Bioinformatics for the future of immunology. Retrieved from https://www.immport.org/immportWeb/queryref/geneListSummary.do

COPE. Retrieved from http://www.copewithcytokines.org/cope.cgi

Santamaria P (2003). Cytokines and chemokines in autoimmune disease: an overview. Adv Exp Med Biol, 520, 1-7.

Edit history

Date	Action	Author
2011-05-12	Created	Garapati P V
2011-05-22	Edited	Ray KP, Jupe S, Garapati P V
2011-05-22	Authored	Ray KP, Jupe S, Garapati P V
2011-05-29	Reviewed	Abdul-Sater AA, Schindler C, Pinteaux E
2018-05-24	Modified	Schmidt EE

Elements found in this pathway

Input	UniProt Id	Input	UniProt Id	Input	UniProt Id
CCL3	P10147	CDKN1A	P38936	DUSP4	Q13115
DUSP6	Q16828	EGR1	P18146	FOXO3	O43524
HMOX1	P09601	ISG20	Q96AZ6	JUN	P05412

Input	UniProt Id	Input	UniProt Id	Input	UniProt Id
MCL1	Q07820	MT2A	P02795	SQSTM1	Q13501

Input	Ensembl Id	Input	Ensembl Id	Input	Ensembl Id
CCL3	ENSG00000277632	CDKN1A	ENSG00000124762	EGR1	ENSG00000120738
FOXO3	ENSG00000118689	HMOX1	ENSG00000100292	ISG20	ENSG00000172183
MCL1	ENSG00000143384	MT2A	ENSG00000125148		

2. Signaling by Interleukins (R-HSA-449147)

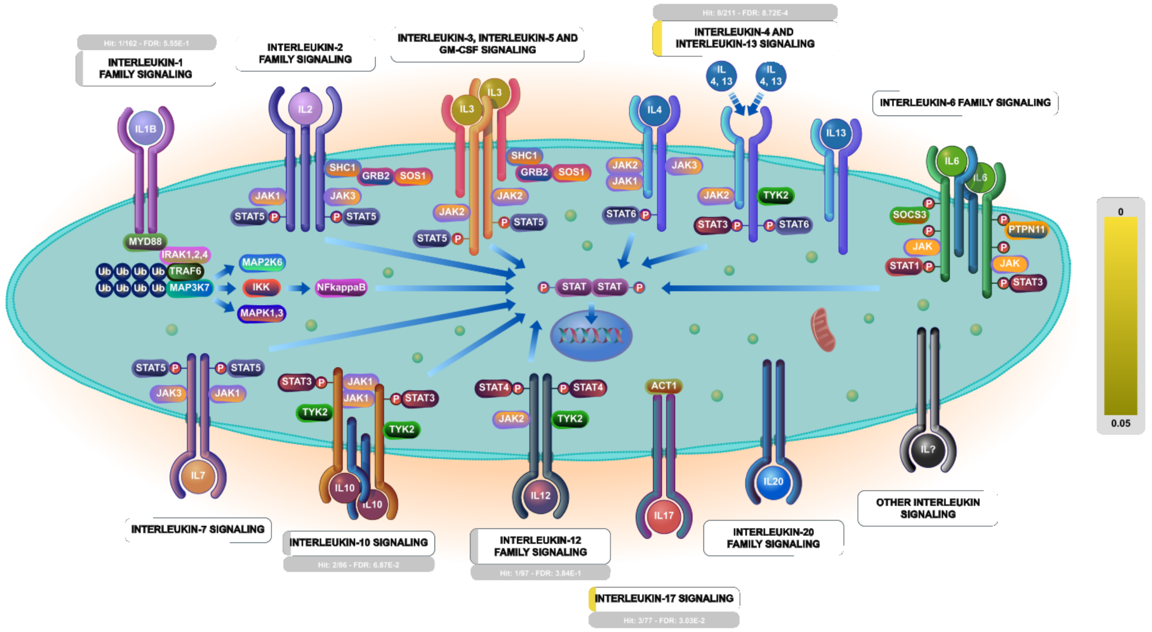

Cellular compartments: plasma membrane.

Interleukins are low molecular weight proteins that bind to cell surface receptors and act in an autocrine and/or paracrine fashion. They were first identified as factors produced by leukocytes but are now known to be produced by many other cells throughout the body. They have pleiotropic effects on cells which bind them, impacting processes such as tissue growth and repair, hematopoietic homeostasis, and multiple levels of the host defense against pathogens where they are an essential part of the immune system.

References

Vosshenrich CA & Di Santo JP (2002). Interleukin signaling. Curr Biol, 12, R760-3.

Dinarello CA (2009). Immunological and inflammatory functions of the interleukin-1 family. Annu Rev Immunol, 27, 519-50.

Akdis M, Aab A, Altunbulakli C, Azkur K, Costa RA, Crameri R, ... Akdis CA (2016). Interleukins (from IL-1 to IL-38), interferons, transforming growth factor , and TNF-: Receptors, functions, and roles in diseases. J. Allergy Clin. Immunol., 138, 984-1010.

Edit history

Date	Action	Author
2009-11-27	Created	Jupe S
2010-05-17	Reviewed	Pinteaux E
2010-05-17	Authored	Ray KP
2010-05-26	Edited	Jupe S
2018-05-24	Modified	Schmidt EE

Elements found in this pathway

Input	UniProt Id	Input	UniProt Id	Input	UniProt Id
CCL3	P10147	CDKN1A	P38936	DUSP4	Q13115
DUSP6	Q16828	FOXO3	O43524	HMOX1	P09601
JUN	P05412	MCL1	Q07820	SQSTM1	Q13501

Input	Ensembl Id	Input	Ensembl Id	Input	Ensembl Id
CCL3	ENSG00000277632	CDKN1A	ENSG00000124762	FOXO3	ENSG00000118689
HMOX1	ENSG00000100292	MCL1	ENSG00000143384		

3. Transcriptional activation of p53 responsive genes (R-HSA-69560)

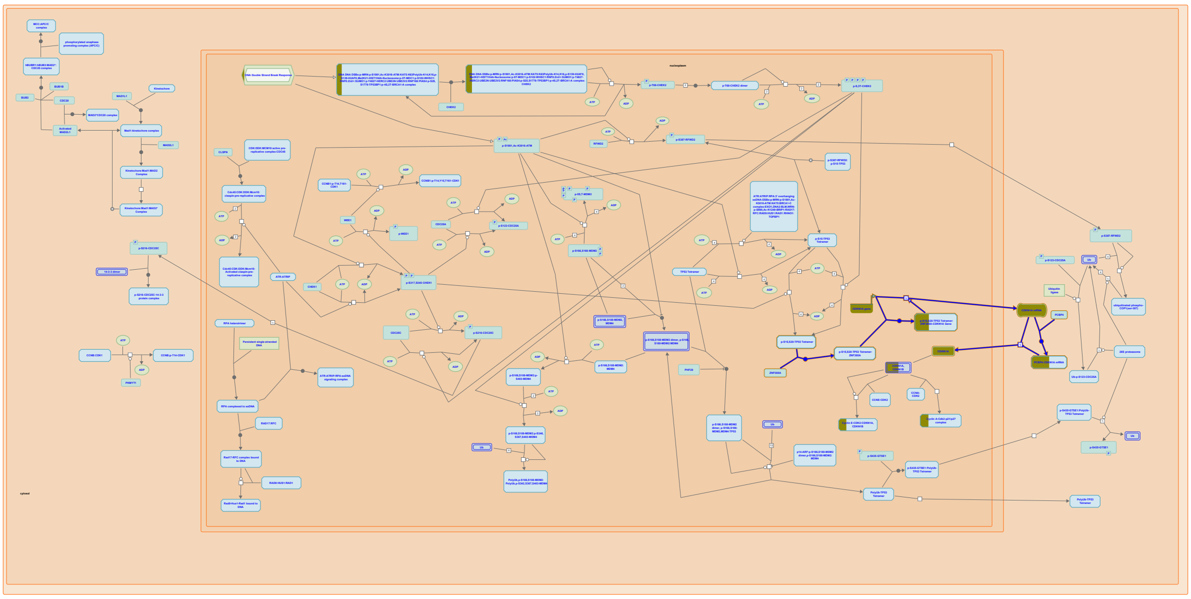

p53 causes G1 arrest by inducing the expression of a cell cycle inhibitor, p21 (El-Deiry et al, 1993; Harper et al, 1993; Xiong et al, 1993). P21 binds and inactivates Cyclin-Cdk complexes that mediate G1/S progression, resulting in lack of phosphorylation of Rb, E2F sequestration and cell cycle arrest at the G1/S transition. Mice with a homozygous deletion of p21 gene are deficient in their ability to undergo a G1/S arrest in response to DNA damage (Deng et al, 1995).

References

el-Deiry WS, Tokino T, Velculescu VE, Levy DB, Parsons R, Trent JM, ... Vogelstein B (1993). WAF1, a potential mediator of p53 tumor suppression. Cell, 75, 817-25.

Harper JW, Adami GR, Wei N, Keyomarsi K & Elledge SJ (1993). The p21 Cdk-interacting protein Cip1 is a potent inhibitor of G1 cyclin-dependent kinases. Cell, 75, 805-16.

Xiong Y, Hannon GJ, Zhang H, Casso D, Kobayashi R & Beach D (1994). p21 is a universal inhibitor of cyclin kinases. Nature, 366, 701-4.

Deng C, Zhang P, Harper JW, Elledge SJ & Leder P (1995). Mice lacking p21CIP1/WAF1 undergo normal development, but are defective in G1 checkpoint control. Cell, 82, 675-84.

Edit history

Date	Action	Author
2003-06-05	Created	Khanna KK
2018-05-23	Modified	Schmidt EE

Elements found in this pathway

Input	UniProt Id	Input	UniProt Id	Input	UniProt Id
CDKN1A	P38936				

Input	Ensembl Id	Input	Ensembl Id	Input	Ensembl Id
CDKN1A	ENST00000244741, ENSG00000124762				

4. Transcriptional activation of cell cycle inhibitor p21 (R-HSA-69895)

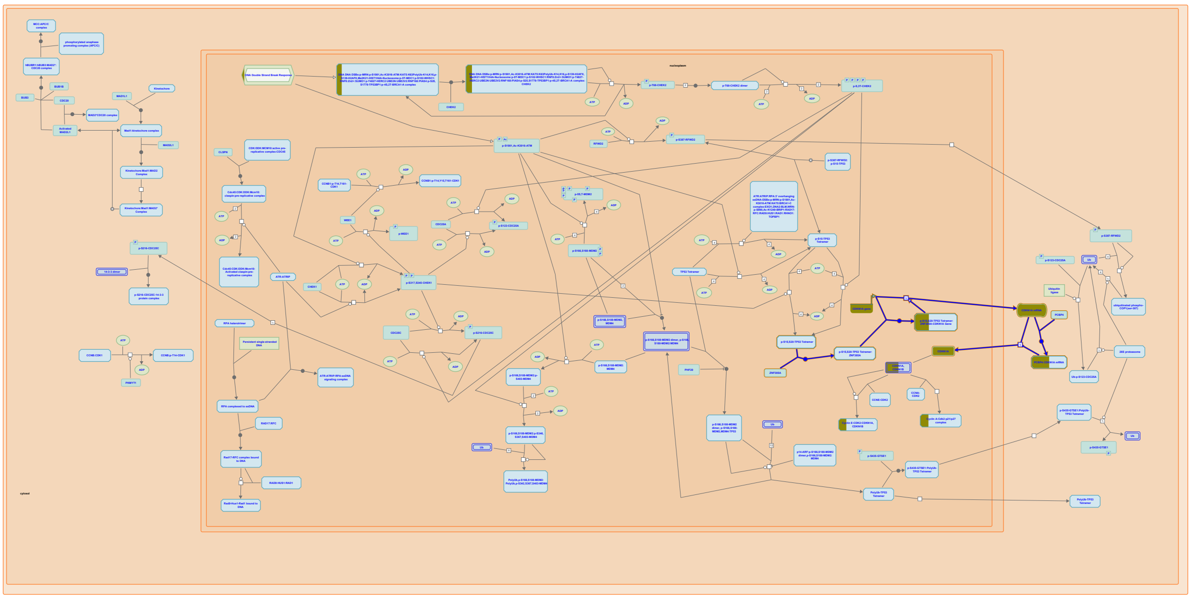

reactome

Both p53-independent and p53-dependent mechanisms of induction of p21 mRNA have been demonstrated. p21 is transcriptionally activated by p53 after DNA damage (el-Deiry et al., 1993).

References

el-Deiry WS, Tokino T, Velculescu VE, Levy DB, Parsons R, Trent JM, ... Vogelstein B (1993). WAF1, a potential mediator of p53 tumor suppression. Cell, 75, 817-25.

Edit history

Date	Action	Author
2003-06-05	Created	Khanna KK
2018-06-04	Modified	Croft D

Elements found in this pathway

Input	UniProt Id	Input	UniProt Id	Input	UniProt Id
CDKN1A	P38936				

Input	Ensembl Id	Input	Ensembl Id	Input	Ensembl Id
CDKN1A	ENST00000244741, ENSG00000124762				

5. Interleukin-4 and Interleukin-13 signaling (R-HSA-6785807)

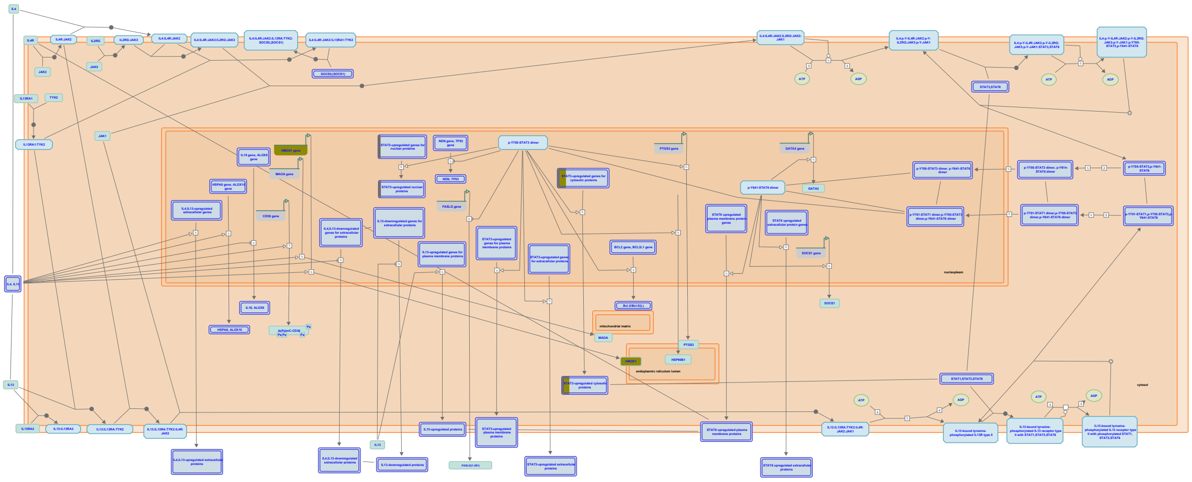

Interleukin-4 (IL4) is a principal regulatory cytokine during the immune response, crucially important in allergy and asthma (Nelms et al. 1999). When resting T cells are antigen-activated and expand in response to Interleukin-2 (IL2), they can differentiate as Type 1 (Th1) or Type 2 (Th2) T helper cells. The outcome is influenced by IL4. Th2 cells secrete IL4, which both stimulates Th2 in an autocrine fashion and acts as a potent B cell growth factor to promote humoral immunity (Nelms et al. 1999).

Interleukin-13 (IL13) is an immunoregulatory cytokine secreted predominantly by activated Th2 cells. It is a key mediator in the pathogenesis of allergic inflammation. IL13 shares many functional properties with IL4, stemming from the fact that they share a common receptor subunit. IL13 receptors are expressed on human B cells, basophils, eosinophils, mast cells, endothelial cells, fibroblasts, monocytes, macrophages, respiratory epithelial cells, and smooth muscle cells, but unlike IL4, not T cells. Thus IL13 does not appear to be important in the initial differentiation of CD4 T cells into Th2 cells, rather it is important in the effector phase of allergic inflammation (Hershey et al. 2003).

IL4 and IL13 induce “alternative activation” of macrophages, inducing an anti-inflammatory phenotype by signaling through IL4R alpha in a STAT6 dependent manner. This signaling plays an important role in the Th2 response, mediating anti-parasitic effects and aiding wound healing (Gordon & Martinez 2010, Loke et al. 2002)

There are two types of IL4 receptor complex (Andrews et al. 2006). Type I IL4R (IL4R1) is predominantly expressed on the surface of hematopoietic cells and consists of IL4R and IL2RG, the common gamma chain. Type II IL4R (IL4R2) is predominantly expressed on the surface of nonhematopoietic cells, it consists of IL4R and IL13RA1 and is also the type II receptor for IL13. (Obiri et al. 1995, Aman et al. 1996, Hilton et al. 1996, Miloux et al. 1997, Zhang et al. 1997). The second receptor for IL13 consists of IL4R and Interleukin-13 receptor alpha 2 (IL13RA2), sometimes called Interleukin-13 binding protein (IL13BP). It has a high affinity receptor for IL13 (Kd = 250 pmol/L) but is not sufficient to render cells responsive to IL13, even in the presence of IL4R (Donaldson et al. 1998). It is reported to exist in soluble form (Zhang et al. 1997) and when overexpressed reduces JAK-STAT signaling (Kawakami et al. 2001). It's function may be to prevent IL13 signalling via the functional IL4R:IL13RA1 receptor. IL13RA2 is overexpressed and enhances cell invasion in some human cancers (Joshi & Puri 2012).

The first step in the formation of IL4R1 (IL4:IL4R:IL2RB) is the binding of IL4 with IL4R (Hoffman et al. 1995, Shen et al. 1996, Hage et al. 1999). This is also the first step in formation of IL4R2 (IL4:IL4R:IL13RA1). After the initial binding of IL4 and IL4R, IL2RB binds (LaPorte et al. 2008), to form IL4R1. Alternatively, IL13RA1 binds, forming IL4R2. In contrast, the type II IL13 complex (IL13R2) forms with IL13 first binding to IL13RA1 followed by recruitment of IL4R (Wang et al. 2009).

Crystal structures of the IL4:IL4R:IL2RG, IL4:IL4R:IL13RA1 and IL13:IL4R:IL13RA1 complexes have been determined (LaPorte et al. 2008). Consistent with these structures, in monocytes IL4R is tyrosine phosphorylated in response to both IL4 and IL13 (Roy et al. 2002, Gordon & Martinez 2010) while IL13RA1 phosphorylation is induced only by IL13 (Roy et al. 2002, LaPorte et al. 2008) and IL2RG phosphorylation is induced only by IL4 (Roy et al. 2002).

Both IL4 receptor complexes signal through Jak/STAT cascades. IL4R is constitutively-associated with JAK2 (Roy et al. 2002) and associates with JAK1 following binding of IL4 (Yin et al. 1994) or IL13 (Roy et al. 2002). IL2RG constitutively associates with JAK3 (Boussiotis et al. 1994, Russell et al. 1994). IL13RA1 constitutively associates with TYK2 (Umeshita-Suyama et al. 2000, Roy et al. 2002, LaPorte et al. 2008, Bhattacharjee et al. 2013).

IL4 binding to IL4R1 leads to phosphorylation of JAK1 (but not JAK2) and STAT6 activation (Takeda et al. 1994, Ratthe et al. 2007, Bhattacharjee et al. 2013).

IL13 binding increases activating tyrosine-99 phosphorylation of IL13RA1 but not that of IL2RG. IL4 binding to IL2RG leads to its tyrosine phosphorylation (Roy et al. 2002). IL13 binding to IL4R2 leads to TYK2 and JAK2 (but not JAK1) phosphorylation (Roy & Cathcart 1998, Roy et al. 2002).

Phosphorylated TYK2 binds and phosphorylates STAT6 and possibly STAT1 (Bhattacharjee et al. 2013).

A second mechanism of signal transduction activated by IL4 and IL13 leads to the insulin receptor substrate (IRS) family (Kelly-Welch et al. 2003). IL4R1 associates with insulin receptor substrate 2 and activates the PI3K/Akt and Ras/MEK/Erk pathways involved in cell proliferation, survival and translational control. IL4R2 does not associate with insulin receptor substrate 2 and consequently the PI3K/Akt and Ras/MEK/Erk pathways are not activated (Busch-Dienstfertig & González-Rodríguez 2013).

References

Nelms K, Keegan AD, Zamorano J, Ryan JJ & Paul WE (1999). The IL-4 receptor: signaling mechanisms and biologic functions. Annu. Rev. Immunol., 17, 701-38.

Hershey GK (2003). IL-13 receptors and signaling pathways: an evolving web. J. Allergy Clin. Immunol., 111, 677-90; quiz 691.

Edit history

Date	Action	Author
2015-07-01	Authored	Jupe S
2015-07-01	Created	Jupe S
2016-09-02	Edited	Jupe S
2016-09-02	Reviewed	Leibovich SJ

Date	Action	Author
2018-06-04	Modified	Croft D

Elements found in this pathway

Input	UniProt Id	Input	UniProt Id	Input	UniProt Id
CDKN1A	P38936	FOXO3	O43524	HMOX1	P09601
MCL1	Q07820				

Input	Ensembl Id	Input	Ensembl Id	Input	Ensembl Id
CDKN1A	ENSG00000124762	FOXO3	ENSG00000118689	HMOX1	ENSG00000100292
MCL1	ENSG00000143384				

6. RAF-independent MAPK1/3 activation (R-HSA-112409)

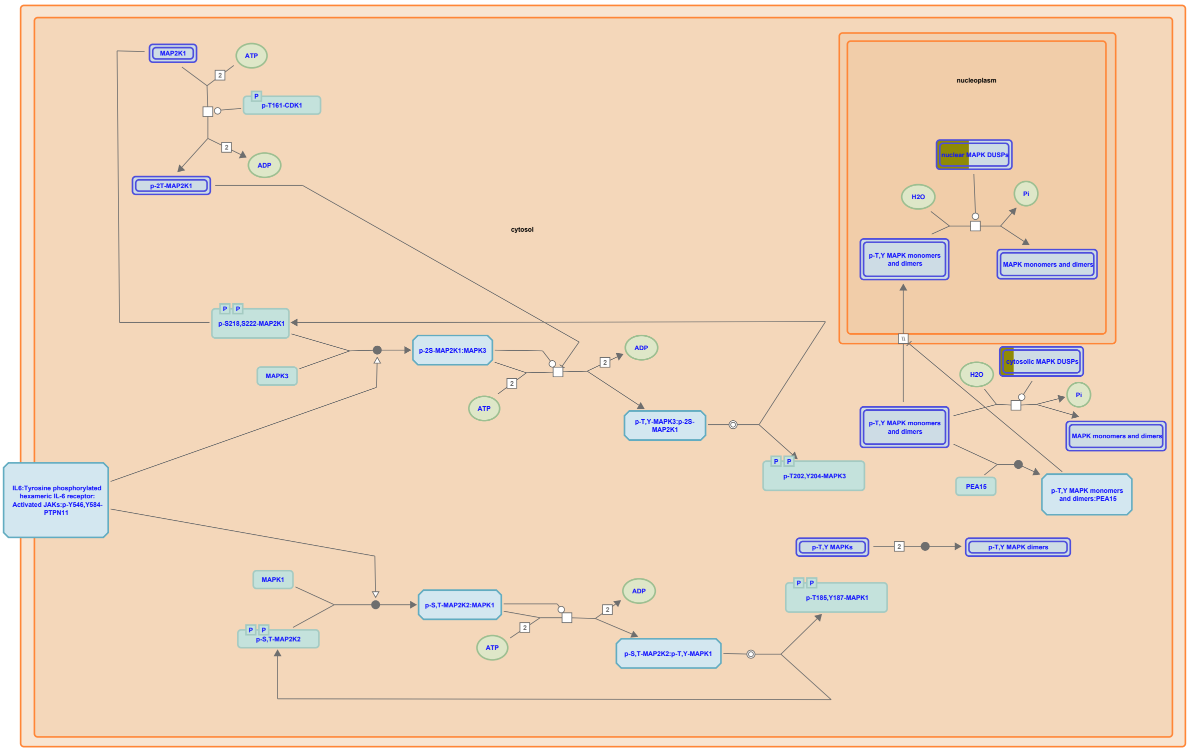

Cellular compartments: cytosol, nucleoplasm.

Depending upon the stimulus and cell type mitogen-activated protein kinases (MAPK) signaling pathway can transmit signals to regulate many different biological processes by virtue of their ability to target multiple effector proteins (Kyriakis JM & Avruch J 2012; Yoon and Seger 2006; Shaul YD & Seger R 2007; Arthur JS & Ley SC 2013). In particular, the extracellular signal-regulated kinases MAPK3(ERK1) and MAPK1 (ERK2) are involved in diverse cellular processes such as proliferation, differentiation, regulation of inflammatory responses, cytoskeletal remodeling, cell motility and invasion through the increase of matrix metalloproteinase production (Viala E & Pouyssegur J 2004; Hsu MC et al. 2006; Dawson CW et al.2008; Kuriakose T et al. 2014).The canonical RAF:MAP2K:MAPK1/3 cascade is stimulated by various extracellular stimuli including hormones, cytokines, growth factors, heat shock and UV irradiation triggering the GEF-mediated activation of RAS at the plasma membrane and leading to the activation of the RAF MAP3 kinases. However, many physiological and pathological stimuli have been found to activate MAPK1/3 independently of RAF and RAS (Dawson CW et al. 2008; Wang J et al. 2009; Kuriakose T et al. 2014). For example, AMP-activated protein kinase (AMPK), but not RAF1, was reported to regulate MAP2K1/2 and MAPK1/3 (MEK and ERK) activation in rat hepatoma H4IIE and human erythroleukemia K562 cells in response to autophagy stimuli (Wang J et al. 2009). Tumor progression locus 2 (TPL2, also known as MAP3K8 and COT) is another MAP3 kinase which promotes MAPK1/3 (ERK)-regulated immune responses downstream of toll-like receptors (TLR), TNF receptor and IL1beta signaling pathways (Gantke T et al. 2011).

In response to stimuli the cell surface receptors transmit signals inducing MAP3 kinases, e.g., TPL2, MEKK1, which in turn phosphorylate MAP2Ks (MEK1/2). MAP2K then phosphorylate and activate the MAPK1/3 (ERK1 and ERK2 MAPKs). Activated MAPK1/3 phosphorylate and regulate the activities of an ever growing pool of substrates that are estimated to comprise over 160 proteins (Yoon and Seger 2006). The majority of ERK substrates are nuclear proteins, but others are found in the cytoplasm and other organelles. Activated MAPK1/3 can translocate to the nucleus, where they phosphorylate and regulate various transcription factors, such as Ets family transcription factors (e.g., ELK1), ultimately leading to changes in gene expression (Zuber J et al. 2000).

References

Arthur JS & Ley SC (2013). Mitogen-activated protein kinases in innate immunity. Nat. Rev. Immunol., 13, 679-92.

Roskoski R Jr (2012). ERK1/2 MAP kinases: structure, function, and regulation. Pharmacol. Res., 66, 105-43.

Gantke T, Sriskantharajah S & Ley SC (2011). Regulation and function of TPL-2, an I?B kinase-regulated MAP kinase kinase kinase. Cell Res., 21, 131-45.

Edit history

Date	Action	Author
2004-04-29	Created	Charalambous M
2007-11-08	Reviewed	Greene LA
2018-06-04	Modified	Croft D

Elements found in this pathway

Input	UniProt Id	Input	UniProt Id	Input	UniProt Id
DUSP1	P28562	DUSP4	Q13115	DUSP5	Q16690
DUSP6	Q16828				

7. Negative regulation of MAPK pathway (R-HSA-5675221)

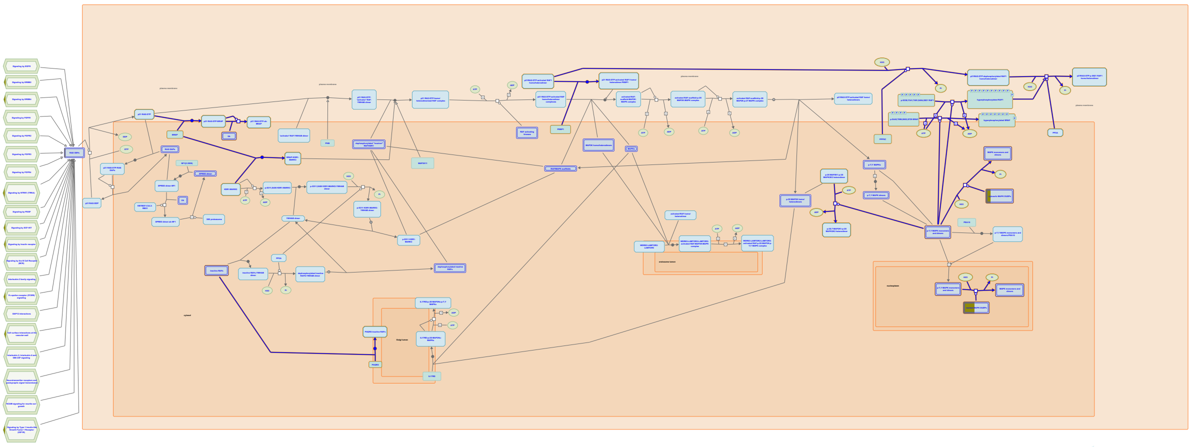

The duration and extent of activated MAPK signaling is regulated at many levels through mechanisms that include phosphorylation and dephosphorylation, changes to protein interacting partners and subcellular localization (reviewed in Matallanas et al, 2011).

Activated RAF proteins are subject to MAPK-dependent phosphorylation that promotes the subsequent dephosphorylation of the activation loop and NtA region, terminating RAF kinase activity. This dephosphorylation, catalyzed by PP2A and PP5, primes the RAF proteins for PKA or AKT-mediated phosphorylation of residues S259 and S621, restoring the 14-3-3 binding sites and returning the RAF proteins to the inactive state (von Kriegsheim et al, 2006; Dougherty et al, 2005; reviewed in Matallanas et al, 2011). The phosphorylated RAF1 NtA is also subject to additional regulation through binding to the PEBP1 protein, which promotes its dissociation from MAP2K substrates (Shin et al, 2009).

Activated MAPK proteins also phosphorylate T292 of MAP2K1; this phosphorylation limits the activity of MAP2K1, and indirectly affects MAP2K2 activity through by modulating the activity of the MAP2K heterodimer (Catalanotti et al, 2009; reviewed in Matallanas et al, 2011).

Dephosphorylation of MAPKs by the dual specificity MAPK phosphatases (DUSPs) plays a key role in limiting the extent of pathway activation (Owens et al, 2007; reviewed in Roskoski, 2012b). Class I DUSPs are localized in the nucleus and are induced by activation of the MAPK pathway, establishing a negative feedback loop, while class II DUSPs dephosphorylate cytoplasmic MAPKs (reviewed in Rososki, 2012b).

MAPK signaling is also regulated by the RAS GAP-mediated stimulation of intrinsic RAS GTPase activity which returns RAS to the inactive, GDP bound state (reviewed in King et al, 2013).

References

Shin SY, Rath O, Choo SM, Fee F, McFerran B, Kolch W & Cho KH (2009). Positive- and negative-feedback regulations coordinate the dynamic behavior of the Ras-Raf-MEK-ERK signal transduction pathway. J. Cell. Sci., 122, 425-35.

Matallanas D, Birtwistle M, Romano D, Zebisch A, Rauch J, von Kriegsheim A & Kolch W (2011). Raf family kinases: old dogs have learned new tricks. Genes Cancer, 2, 232-60.

von Kriegsheim A, Pitt A, Grindlay GJ, Kolch W & Dhillon AS (2006). Regulation of the Raf-MEK-ERK pathway by protein phosphatase 5. Nat. Cell Biol., 8, 1011-6.

Dougherty MK, Müller J, Ritt DA, Zhou M, Zhou XZ, Copeland TD, ... Morrison DK (2005). Regulation of Raf-1 by direct feedback phosphorylation. Mol. Cell, 17, 215-24.

Catalanotti F, Reyes G, Jesenberger V, Galabova-Kovacs G, de Matos Simoes R, Carugo O & Baccarini M (2009). A Mek1-Mek2 heterodimer determines the strength and duration of the Erk signal. Nat. Struct. Mol. Biol., 16, 294-303.

Edit history

Date	Action	Author
2015-02-12	Edited	Rothfels K
2015-02-12	Authored	Rothfels K
2015-02-15	Created	Rothfels K
2015-04-29	Reviewed	Roskoski R Jr
2018-06-04	Modified	Croft D

Elements found in this pathway

Input	UniProt Id	Input	UniProt Id	Input	UniProt Id
DUSP1	P28562	DUSP4	Q13115	DUSP5	Q16690
DUSP6	Q16828				

8. TP53 Regulates Transcription of Genes Involved in G1 Cell Cycle Arrest (R-HSA-6804116)

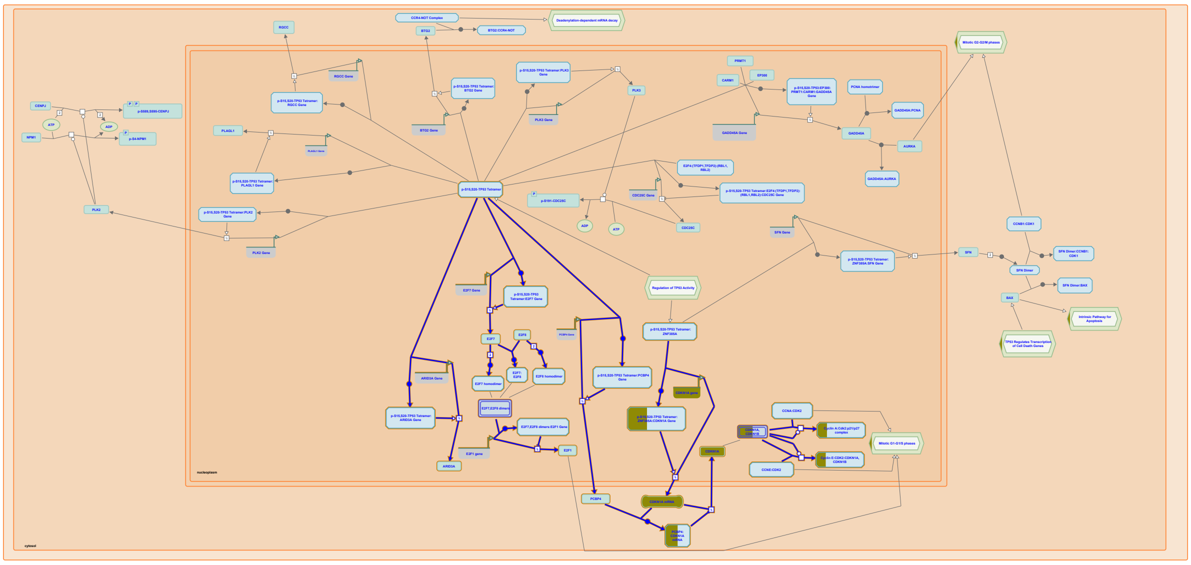

The most prominent TP53 target involved in G1 arrest is the inhibitor of cyclin-dependent kinases CDKN1A (p21). CDKN1A is one of the earliest genes induced by TP53 (El-Deiry et al. 1993). CDKN1A binds and inactivates CDK2 in complex with cyclin A (CCNA) or E (CCNE), thus preventing G1/S transition (Harper et al. 1993). Considering its impact on the cell cycle outcome, CDKN1A expression levels are tightly regulated. For instance, under prolonged stress, TP53 can induce the transcription of an RNA binding protein PCBP4, which can bind and destabilize CDKN1A mRNA, thus alleviating G1 arrest and directing the affected cell towards G2 arrest and, possibly, apoptosis (Zhu and Chen 2000, Scoumanne et al. 2011). Expression of E2F7 is directly induced by TP53. E2F7 contributes to G1 cell cycle arrest by repressing transcription of E2F1, a transcription factor that promotes expression of many genes needed for G1/S transition (Aksoy et al. 2012, Carvajal et al. 2012). ARID3A is a direct transcriptional target of TP53 (Ma et al. 2003) that may promote G1 arrest by co-operating with TP53 in induction of CDKN1A transcription (Lestari et al. 2012). However, ARID3A may also promote G1/S transition by stimulating transcriptional activity of E2F1 (Suzuki et al. 1998, Peeper et al. 2002).

TP53 has co-factors that are key determinants of transcriptional selectivity within the p53 network. For instance, the zinc finger transcription factor ZNF385A (HZF) is a direct transcriptional target of TP53 that can form a complex with TP53 and facilitate TP53-mediated induction of CDKN1A, strongly favouring cell cycle arrest over apoptosis (Das et al. 2007).

References

el-Deiry WS, Tokino T, Velculescu VE, Levy DB, Parsons R, Trent JM, ... Vogelstein B (1993). WAF1, a potential mediator of p53 tumor suppression. Cell, 75, 817-25.

Harper JW, Adami GR, Wei N, Keyomarsi K & Elledge SJ (1993). The p21 Cdk-interacting protein Cip1 is a potent inhibitor of G1 cyclin-dependent kinases. Cell, 75, 805-16.

Zhu J & Chen X (2000). MCG10, a novel p53 target gene that encodes a KH domain RNA-binding protein, is capable of inducing apoptosis and cell cycle arrest in G(2)-M. Mol. Cell. Biol., 20, 5602-18.

Scoumanne A, Cho SJ, Zhang J & Chen X (2011). The cyclin-dependent kinase inhibitor p21 is regulated by RNA-binding protein PCBP4 via mRNA stability. Nucleic Acids Res., 39, 213-24.

Aksoy O, Chicas A, Zeng T, Zhao Z, McCurrach M, Wang X & Lowe SW (2012). The atypical E2F family member E2F7 couples the p53 and RB pathways during cellular senescence. Genes Dev., 26, 1546-57.

Edit history

Date	Action	Author
2015-10-08	Created	Orlic-Milacic M
2015-10-14	Edited	Orlic-Milacic M
2015-10-14	Authored	Orlic-Milacic M
2016-02-04	Reviewed	Zaccara S, Inga A
2017-01-03	Revised	Orlic-Milacic M
2018-06-04	Modified	Croft D

Elements found in this pathway

Input	UniProt Id	Input	UniProt Id	Input	UniProt Id
CDKN1A	P38936				

Input	Ensembl Id	Input	Ensembl Id	Input	Ensembl Id
CDKN1A	ENST00000244741, ENSG00000124762				

9. Activation of NOXA and translocation to mitochondria (R-HSA-111448)

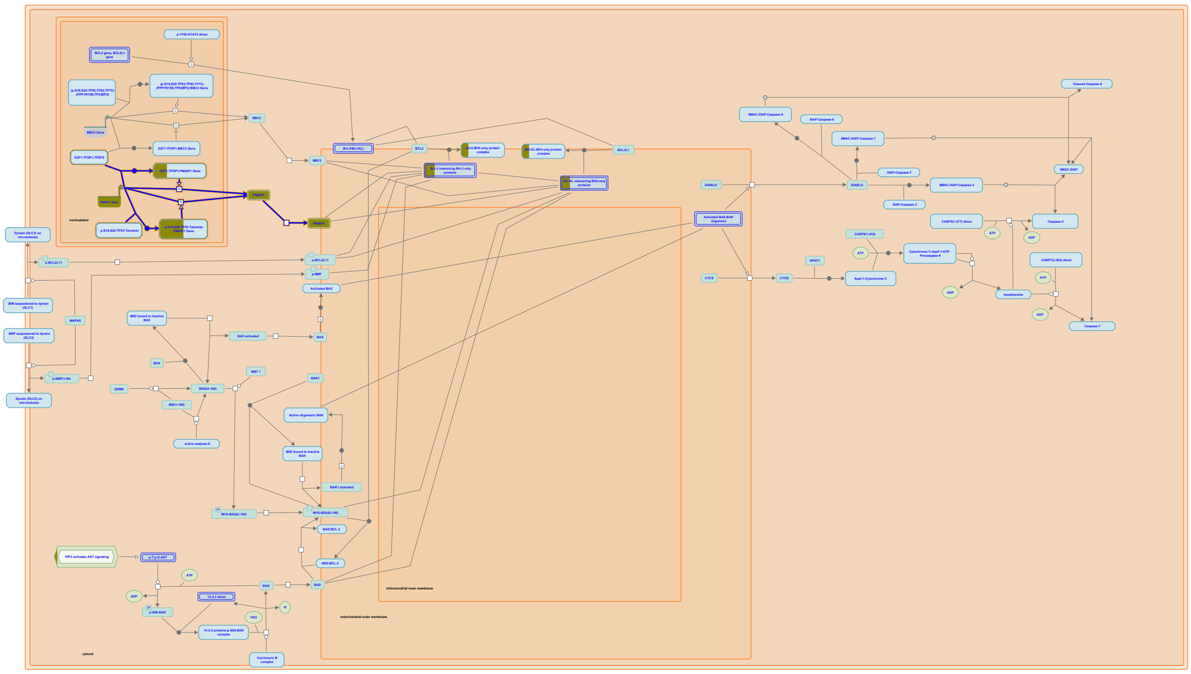

Cellular compartments: cytosol.

NOXA is transactivated in a p53-dependent manner and by E2F1. Activated NOXA is translocated to mitochondria.

References

Li CQ, Robles AI, Hanigan CL, Hofseth LJ, Trudel LJ, Harris CC & Wogan GN (2004). Apoptotic signaling pathways induced by nitric oxide in human lymphoblastoid cells expressing wild-type or mutant p53. Cancer Res, 64, 3022-9.

Hershko T & Ginsberg D (2004). Up-regulation of Bcl-2 homology 3 (BH3)-only proteins by E2F1 mediates apoptosis. J Biol Chem, 279, 8627-34.

Edit history

Date	Action	Author
2004-08-10	Created	Tsujimoto Y, Hardwick JM
2018-05-23	Modified	Schmidt EE

Elements found in this pathway

Input	UniProt Id	Input	UniProt Id	Input	UniProt Id
PMAIP1	Q13794				

Input	Ensembl Id	Input	Ensembl Id	Input	Ensembl Id
PMAIP1	ENSG00000141682				

10. TFAP2 (AP-2) family regulates transcription of cell cycle factors (R-HSA-8866911)

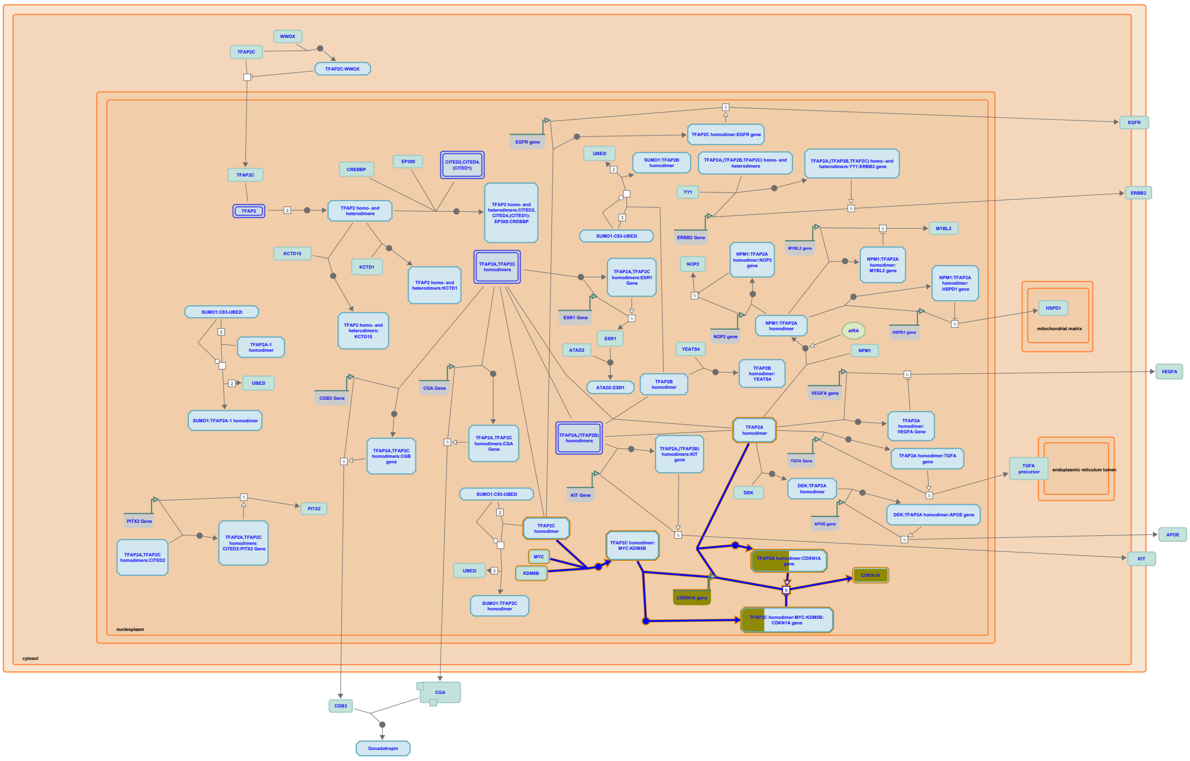

TFAP2A and TFAP2C play opposing roles in transcriptional regulation of the CDKN1A (p21) gene locus. While TFAP2A stimulates transcription of the CDKN1A cyclin-dependent kinase inhibitor (Zeng et al. 1997, Williams et al. 2009, Scibetta et al. 2010), TFAP2C, in cooperation with MYC and histone demethylase KDM5B, represses CDKN1A transcription (Williams et al. 2009, Scibetta et al. 2010, Wong et al. 2012).

References

Zeng YX, Somasundaram K & el-Deiry WS (1997). AP2 inhibits cancer cell growth and activates p21WAF1/CIP1 expression. Nat. Genet., 15, 78-82.

Scibetta AG, Wong PP, Chan KV, Canosa M & Hurst HC (2010). Dual association by TFAP2A during activation of the p21cip/CDKN1A promoter. Cell Cycle, 9, 4525-32.

Williams CM, Scibetta AG, Friedrich JK, Canosa M, Berlato C, Moss CH & Hurst HC (2009). AP-2gamma promotes proliferation in breast tumour cells by direct repression of the CDKN1A gene . EMBO J., 28, 3591-601.

Wong PP, Miranda F, Chan KV, Berlato C, Hurst HC & Scibetta AG (2012). Histone demethylase KDM5B collaborates with TFAP2C and Myc to repress the cell cycle inhibitor p21(cip) (CDKN1A) . Mol. Cell. Biol., 32, 1633-44.

Edit history

Date	Action	Author
2016-03-14	Edited	Orlic-Milacic M
2016-03-14	Authored	Orlic-Milacic M

Date	Action	Author
2016-04-04	Created	Orlic-Milacic M
2016-05-04	Reviewed	Dawid IB, Zarelli VE
2016-05-17	Reviewed	Bogachek MV, Weigel RJ
2018-05-24	Modified	Schmidt EE

Elements found in this pathway

Input	UniProt Id	Input	UniProt Id	Input	UniProt Id
CDKN1A	P38936				

Input	Ensembl Id	Input	Ensembl Id	Input	Ensembl Id
CDKN1A	ENSG00000124762				

11. DNA Damage/Telomere Stress Induced Senescence (R-HSA-2559586)

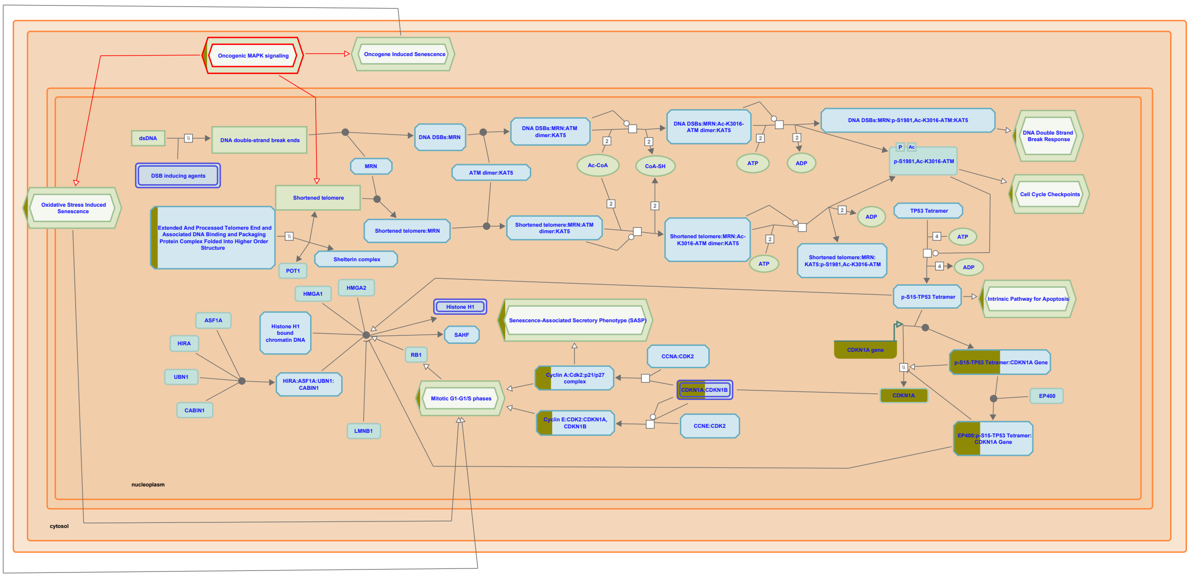

reactome

Reactive oxygen species (ROS), whose concentration increases in senescent cells due to oncogenic RAS-induced mitochondrial dysfunction (Moiseeva et al. 2009) or due to environmental stress, cause DNA damage in the form of double strand breaks (DSBs) (Yu and Anderson 1997). In addition, persistent cell division fueled by oncogenic signaling leads to replicative exhaustion, manifested in critically short telomeres (Harley et al. 1990, Hastie et al. 1990). Shortened telomeres are no longer able to bind the protective shelterin complex (Smogorzewska et al. 2000, de Lange 2005) and are recognized as damaged DNA.

The evolutionarily conserved MRN complex, consisting of MRE11A (MRE11), RAD50 and NBN (NBS1) subunits, binds DSBs (Lee and Paull 2005) and shortened telomeres that are no longer protected by shelterin (Wu et al. 2007). Once bound to the DNA, the MRN complex recruits and activates ATM kinase (Lee and Paull 2005, Wu et al. 2007), leading to phosphorylation of ATM targets, including TP53 (p53) (Banin et al. 1998, Canman et al. 1998, Khanna et al. 1998). TP53, phosphorylated on serine S15 by ATM, binds the CDKN1A (also known as p21, CIP1 or WAF1) promoter and induces CDKN1A transcription (El-Deiry et al. 1993, Karlseder et al. 1999). CDKN1A inhibits the activity of CDK2, leading to G1/S cell cycle arrest (Harper et al. 1993, El-Deiry et al. 1993).

SMURF2 is upregulated in response to telomere attrition in human fibroblasts and induces senecscent phenotype through RB1 and TP53, independently of its role in TGF-beta-1 signaling (Zhang and Cohen 2004). The exact mechanism of SMURF2 involvement is senescence has not been elucidated.

References

Moiseeva O, Bourdeau V, Roux A, Deschênes-Simard X & Ferbeyre G (2009). Mitochondrial dysfunction contributes to oncogene-induced senescence. Mol. Cell. Biol., 29, 4495-507.

Yu TW & Anderson D (1997). Reactive oxygen species-induced DNA damage and its modification: a chemical investigation. Mutat. Res., 379, 201-10.

Harley CB, Futcher AB & Greider CW (1990). Telomeres shorten during ageing of human fibroblasts . Nature, 345, 458-60.

Hastie ND, Dempster M, Dunlop MG, Thompson AM, Green DK & Allshire RC (1990). Telomere reduction in human colorectal carcinoma and with ageing. Nature, 346, 866-8.

de Lange T (2005). Shelterin: the protein complex that shapes and safeguards human telomeres. Genes Dev, 19, 2100-10.

Edit history

Date	Action	Author
2012-11-02	Created	Orlic-Milacic M
2013-07-15	Edited	Matthews L, D'Eustachio P
2013-07-15	Authored	Orlic-Milacic M
2013-09-03	Reviewed	Samarajiwa S
2018-06-04	Modified	Croft D

Elements found in this pathway

Input	UniProt Id	Input	UniProt Id	Input	UniProt Id
CDKN1A	P38936	H2BFS	P57053	HIST1H2BK	O60814

Input	Ensembl Id	Input	Ensembl Id	Input	Ensembl Id
CDKN1A	ENSG00000124762				

12. Neurodegenerative Diseases (R-HSA-8863678)

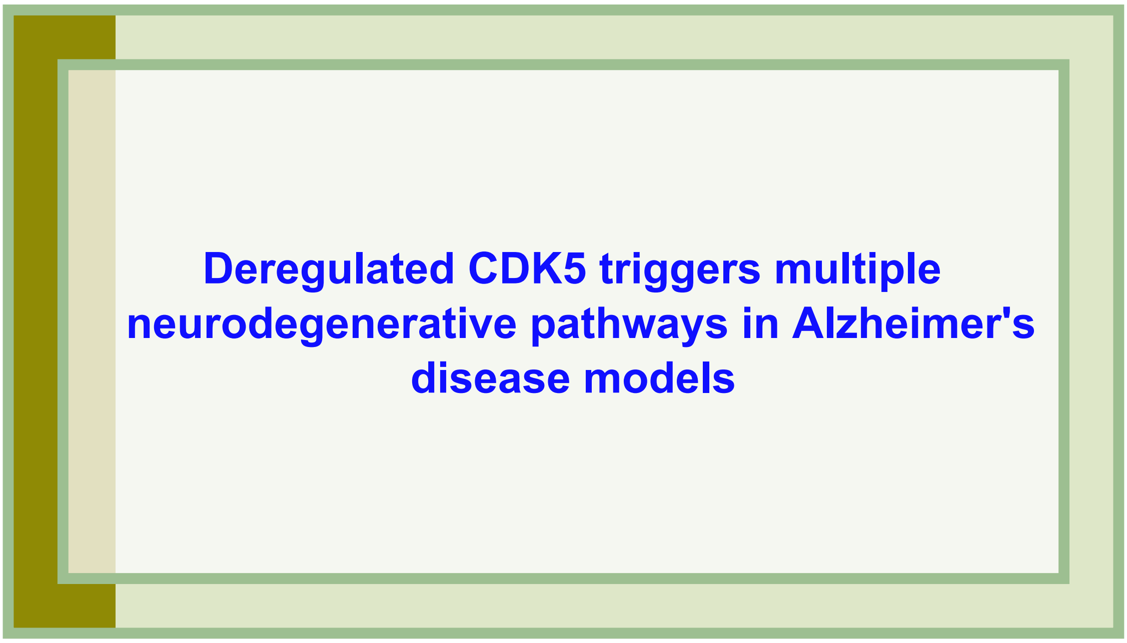

Diseases: neurodegenerative disease.

Neurodegenerative diseases manifest as the progressive dysfunction and loss of neurons, which is frequently accompanied by formation of misfolded protein deposits in the brain. Classification of neurodegenerative diseases is based on clinical symptoms, which depend on the anatomical region affected by neuronal dysfunction, the identity of misfolded proteins and cellular and subcellular pathology.

In Alzheimer's disease (AD), beta-amyloid protein (APP) deposits form in the extracellular space, where they can make plaques, while abnormally phosphorylated tau protein (MAPT) accumulates in neuronal cells.

Beside AD, neuronal and/or glial inclusions of hyperphosphorylated tau are also found in Pick disease (PiD), neurofibrillary tangle-dementia (NFT), primary age-related tauopathy (PART), progressive supranuclear palsy (PSP), corticobasal degeneration (CBD), argyrophilic grain disease (AGD) and globular glial tauopathies (GGT).

In prion disease, such as Creutzfeldt-Jakob disease, deposits of PrP protein are formed mostly in the extracellular and presynaptic space. PrP deposits in neuronal cell bodies are mainly confined to endosomes and lysosomes, which is attributed to neuronal uptake of pathological proteins and intercellular prion spreading.

In Parkinson disease (PD) and dementia with Lewy bodies (DLB), deposits of alpha-synuclein (SNCA) are formed in the cytoplasm of neuronal cell bodies and neurites. In multiple system atrophy (MSA), deposits of alpha-synuclein form in the cytoplasm of glial cells (Papp-Lantos bodies).

Amyotrophic lateral sclerosis (ALS) and frontotemporal lobar degeneration (FTLD) are characterized by ubiquitin-positive cytoplasmic inclusions of TAR DNA-binding protein 43 (TARDBP, commonly known as TDP-43), a protein that normally localizes to the nucleus. Pathological TDP-43 inclusions have been associated with the TDP-43 gene mutations, as well as mutations in several other genes, including C9orf72, GRN, VCP, SQSTM1, DCTN1 and OPTN. TDP-43 inclusions have also been reported in AD, DLB, hippocampal sclerosis (HS) and chronic traumatic encephalopathy.

FUS protein-positive inclusion bodies are found in familial ALS, caused by mutations in the FUS gene, as well as in a small subgroup of FTLD-related diseases. FUS-positive inclusions may be accompanied by FET protein-positive inclusions.

For a detailed review of molecular pathology of neurodegenerative diseases, please refer to Kovacs 2016.

Within this broad domain, the process by which APP-triggered deregulation of CDK5 (cyclin-dependent kinase 5) triggers multiple neurodegenerative pathways associated with Alzheimer's disease has been annotated.

References

Kovacs GG (2016). Molecular Pathological Classification of Neurodegenerative Diseases: Turning towards Precision Medicine. Int J Mol Sci, 17.

Edit history

Date	Action	Author
2016-03-10	Created	Orlic-Milacic M
2016-08-18	Reviewed	D'Eustachio P
2016-08-19	Authored	Orlic-Milacic M
2016-08-20	Modified	Orlic-Milacic M
2016-08-20	Edited	Orlic-Milacic M

Elements found in this pathway

Input	UniProt Id	Input	UniProt Id	Input	UniProt Id
FOXO3	O43524	JUN	P05412	LMNA	P02545-1

13. Deregulated CDK5 triggers multiple neurodegenerative pathways in Alzheimer's disease models (R-HSA-8862803)

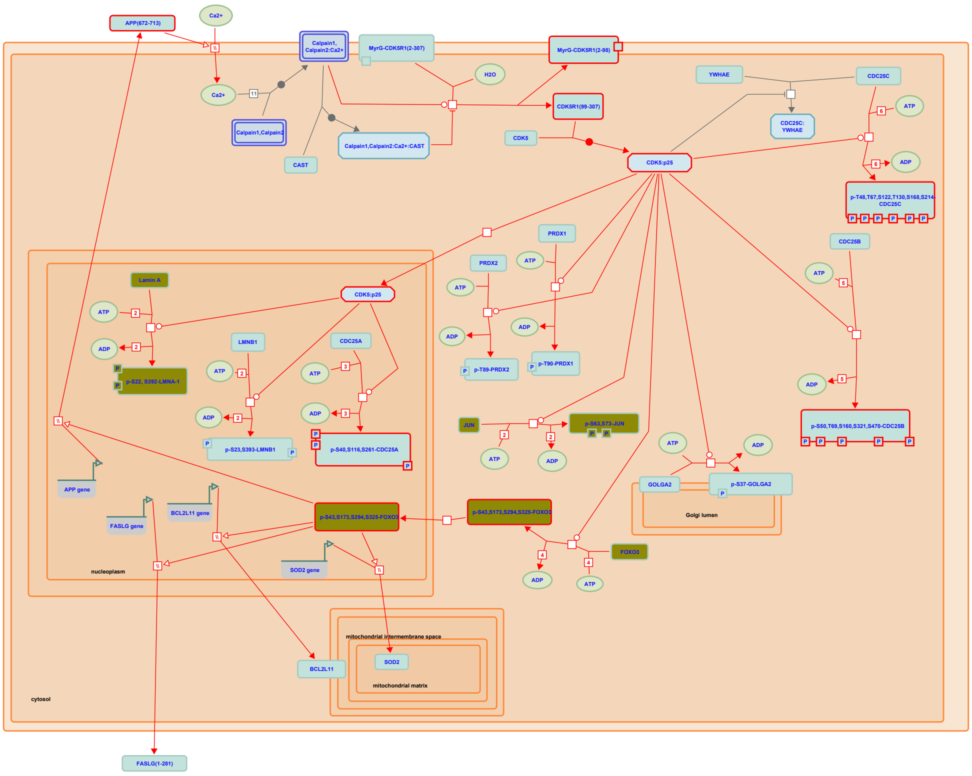

Diseases: Alzheimer's disease.

Post-mitotic neurons do not have an active cell cycle. However, deregulation of Cyclin Dependent Kinase-5 (CDK5) activity in these neurons can aberrantly activate various components of cell cycle leading to neuronal death (Chang et al. 2012). Random activation of cell cycle proteins has been shown to play a key role in the pathogenesis of several neurodegenerative disorders (Yang et al. 2003, Lopes et al. 2009). CDK5 is not activated by the canonical cyclins, but binds to its own specific partners, CDK5R1 and CDK5R2 (aka p35 and p39, respectively) (Tsai et al. 1994, Tang et al. 1995). Expression of p35 is nearly ubiquitous, whereas p39 is largely expressed in the central nervous system. A variety of neurotoxic insults such as beta-amyloid (A-beta), ischemia, excitotoxicity and oxidative stress disrupt the intracellular calcium homeostasis in neurons, thereby leading to the activation of calpain, which cleaves p35 into p25 and p10 (Lee et al. 2000). p25 has a six-fold longer half-life compared to p35 and lacks the membrane anchoring signal, which results in its constitutive activation and mislocalization of the CDK5:p25 complex to the cytoplasm and the nucleus. There, CDK5:p25 is able to access and phosphorylate a variety of atypical targets, triggering a cascade of neurotoxic pathways that culminate in neuronal death. One such neurotoxic pathway involves CDK5-mediated random activation of cell cycle proteins which culminate in neuronal death. Exposure of primary cortical neurons to oligomeric beta-amyloid (1-42) hyper-activates CDK5 due to p25 formation, which in turn phosphorylates CDC25A, CDC25B and CDC25C. CDK5 phosphorylates CDC25A at S40, S116 and S261; CDC25B at S50, T69, S160, S321 and S470; and CDC25C at T48, T67, S122, T130, S168 and S214. CDK5-mediated phosphorylation of CDC25A, CDC25B and CDC25C not only increases their phosphatase activities but also facilitates their release from 14-3-3 inhibitory binding. CDC25A, CDC25B and CDC25C in turn activate CDK1, CDK2 and CDK4 kinases causing neuronal death. Consistent with this mechanism, higher CDC25A, CDC25B and CDC25C activities were observed in human Alzheimer's disease (AD) clinical samples, as compared to age-matched controls. Inhibition of CDC25 isoforms confers neuroprotection to beta-amyloid toxicity, which underscores the contribution of this pathway to AD pathogenesis

References

Chang KH, Vincent F & Shah K (2012). Deregulated Cdk5 triggers aberrant activation of cell cycle kinases and phosphatases inducing neuronal death. J. Cell. Sci., 125, 5124-37.

Yang Y, Mufson EJ & Herrup K (2003). Neuronal cell death is preceded by cell cycle events at all stages of Alzheimer's disease. J. Neurosci., 23, 2557-63.

Lopes JP, Oliveira CR & Agostinho P (2009). Cell cycle re-entry in Alzheimer's disease: a major neuropathological characteristic?. Curr Alzheimer Res, 6, 205-12.

Tsai LH, Delalle I, Caviness VS, Chae T & Harlow E (1994). p35 is a neural-specific regulatory subunit of cyclin-dependent kinase 5. Nature, 371, 419-23.

Tang D, Yeung J, Lee KY, Matsushita M, Matsui H, Tomizawa K, ... Wang JH (1995). An isoform of the neuronal cyclin-dependent kinase 5 (Cdk5) activator. J. Biol. Chem., 270, 26897-903.

Edit history

Date	Action	Author
2016-02-23	Authored	Shah K
2016-03-02	Created	Orlic-Milacic M
2016-05-10	Edited	Orlic-Milacic M
2018-06-04	Modified	Croft D

Elements found in this pathway

Input	UniProt Id	Input	UniProt Id	Input	UniProt Id
FOXO3	O43524	JUN	P05412	LMNA	P02545-1

14. MAPK targets/ Nuclear events mediated by MAP kinases (R-HSA-450282)

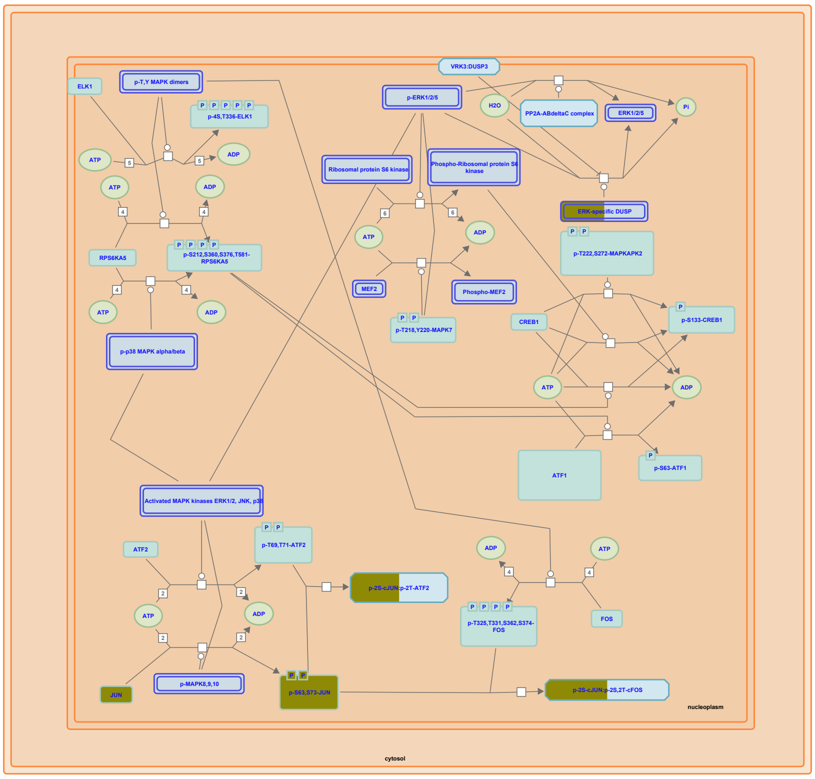

MAPKs are protein kinases that, once activated, phosphorylate their specific cytosolic or nuclear substrates at serine and/or threonine residues. Such phosphorylation events can either positively or negatively regulate substrate, and thus entire signaling cascade activity.

The major cytosolic target of activated ERKs are RSKs (90 kDa Ribosomal protein S6 Kinase). Active RSKs translocates to the nucleus and phosphorylates such factors as c-Fos(on Ser362), SRF (Serum Response Factor) at Ser103, and CREB (Cyclic AMP Response Element-Binding protein) at Ser133. In the nucleus activated ERKs phosphorylate many other targets such as MSKs (Mitogen- and Stress-activated protein kinases), MNK (MAP interacting kinase) and Elk1 (on Serine383 and Serine389). ERK can directly phosphorylate CREB and also AP-1 components c-Jun and c-Fos. Another important target of ERK is NF-KappaB. Recent studies reveals that nuclear pore proteins are direct substrates for ERK (Kosako H et al, 2009). Other ERK nuclear targets include c-Myc, HSF1 (Heat-Shock Factor-1), STAT1/3 (Signal Transducer and Activator of Transcription-1/3), and many more transcription factors.

Activated p38 MAPK is able to phosphorylate a variety of substrates, including transcription factors STAT1, p53, ATF2 (Activating transcription factor 2), MEF2 (Myocyte enhancer factor-2), protein kinases MSK1, MNK, MAPKAPK2/3, death/survival molecules (Bcl2, caspases), and cell cycle control factors (cyclin D1).

JNK, once activated, phosphorylates a range of nuclear substrates, including transcription factors Jun, ATF, Elk1, p53, STAT1/3 and many other factors. JNK has also been shown to directly phosphorylate many nuclear hormone receptors. For example, peroxisome proliferator-activated receptor 1 (PPAR-1) and retinoic acid receptors RXR and RAR are substrates for JNK. Other JNK targets are heterogeneous nuclear ribonucleoprotein K (hnRNP-K) and the Pol I-specific transcription factor TIF-IA, which regulates ribosome synthesis. Other adaptor and scaffold proteins have also been characterized as nonnuclear substrates of JNK.

References

Johnson GL & Lapadat R (2002). Mitogen-activated protein kinase pathways mediated by ERK, JNK, and p38 protein kinases. Science, 298, 1911-2.

Bogoyevitch MA & Kobe B (2006). Uses for JNK: the many and varied substrates of the c-Jun N-terminal kinases. Microbiol Mol Biol Rev, 70, 1061-95.

Yoon S & Seger R (2006). The extracellular signal-regulated kinase: multiple substrates regulate diverse cellular functions. Growth Factors, 24, 21-44.

Edit history

Date	Action	Author
2009-12-16	Authored	Shamovsky V
2009-12-16	Created	Shamovsky V
2010-02-28	Edited	Shamovsky V
2010-02-28	Reviewed	Gillespie ME
2018-05-24	Modified	Schmidt EE

Elements found in this pathway

Input	UniProt Id	Input	UniProt Id	Input	UniProt Id
DUSP4	Q13115	DUSP6	Q16828	JUN	P05412

15. RUNX3 regulates CDKN1A transcription (R-HSA-8941855)

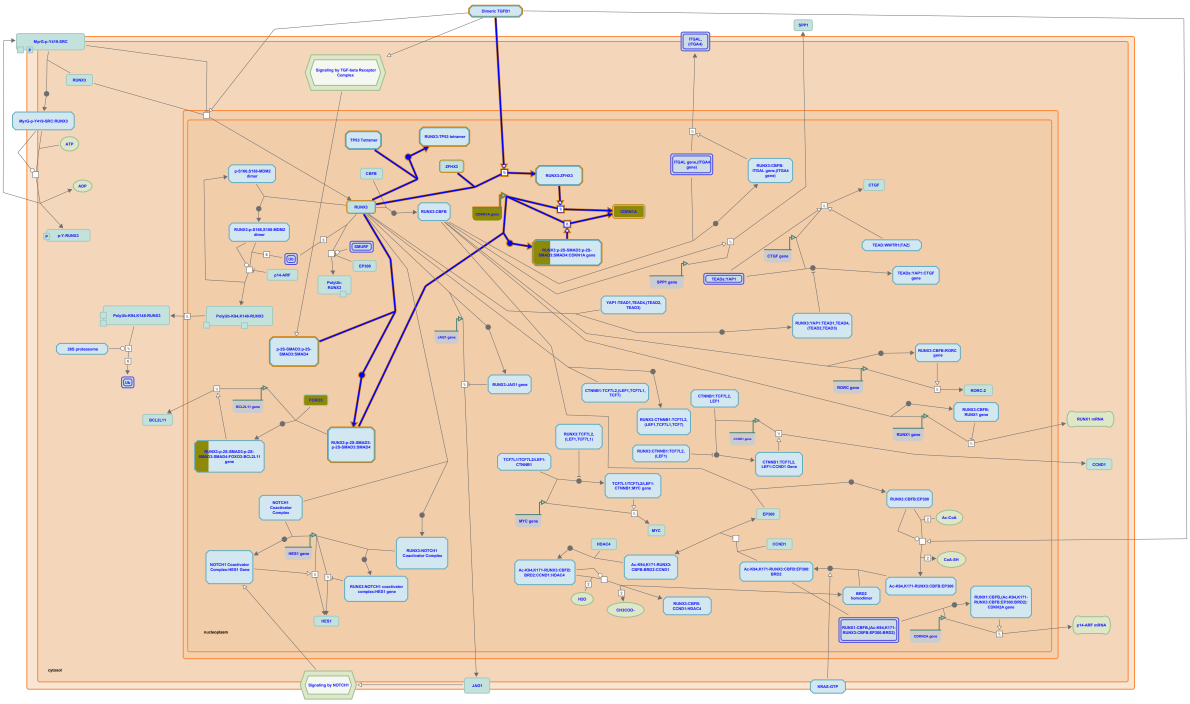

RUNX3 contributes to the upregulation of the CDKN1A (p21) gene transcription in response to TGF-beta (TGFB1) signaling. RUNX3 binds to SMAD3 and SMAD4, and cooperates with the activated SMAD3:SMAD4 complex in transactivation of CDKN1A. Runx3 knockout mice exhibit decreased sensitivity to TGF-beta and develop gastric epithelial hyperplasia (Chi et al. 2005). In response to TGF-beta signaling, the CBFB:RUNX3 complex binds to the tumor suppressor ZFHX3 (ATBF1) and, through an unknown mechanism, this complex positively regulates the CDKN1A transcription (Mabuchi et al. 2010).

In addition, RUNX3 may act as a TP53 co-factor, stimulating TP53-mediated transcription of target genes, including CDKN1A (p21) (Yamada et al. 2010).

References

Chi XZ, Yang JO, Lee KY, Ito K, Sakakura C, Li QL, ... Bae SC (2005). RUNX3 suppresses gastric epithelial cell growth by inducing p21(WAF1/Cip1) expression in cooperation with transforming growth factor {beta}-activated SMAD. Mol. Cell. Biol., 25, 8097-107.

Mabuchi M, Kataoka H, Miura Y, Kim TS, Kawaguchi M, Ebi M, ... Joh T (2010). Tumor suppressor, AT motif binding factor 1 (ATBF1), translocates to the nucleus with runt domain transcription factor 3 (RUNX3) in response to TGF-beta signal transduction. Biochem. Biophys. Res. Commun., 398, 321-5.

Yamada C, Ozaki T, Ando K, Suenaga Y, Inoue K, Ito Y, ... Nakagawara A (2010). RUNX3 modulates DNA damage-mediated phosphorylation of tumor suppressor p53 at Ser-15 and acts as a co-activator for p53. J. Biol. Chem., 285, 16693-703.

Edit history

Date	Action	Author
2016-10-07	Created	Orlic-Milacic M

Date	Action	Author
2016-12-13	Authored	Orlic-Milacic M
2017-01-31	Edited	Orlic-Milacic M
2017-01-31	Reviewed	Ito Y, Chuang LS
2018-06-04	Modified	Croft D

Elements found in this pathway

Input	UniProt Id	Input	UniProt Id	Input	UniProt Id
CDKN1A	P38936				

Input	Ensembl Id	Input	Ensembl Id	Input	Ensembl Id
CDKN1A	ENSG00000124762				

16. Senescence-Associated Secretory Phenotype (SASP) (R-HSA-2559582)

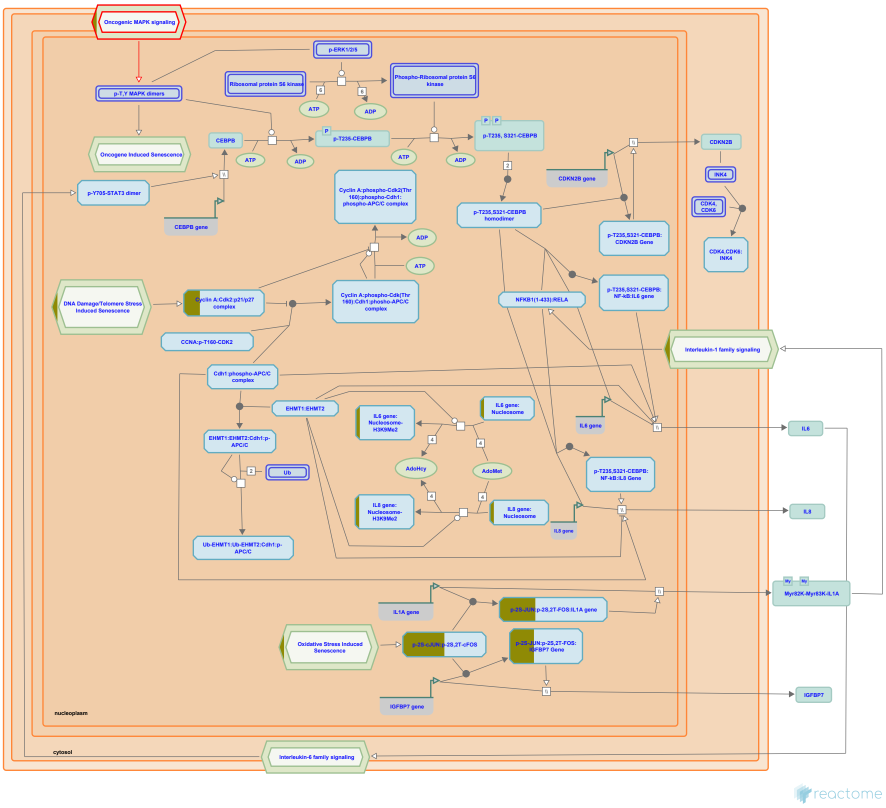

The culture medium of senescent cells in enriched in secreted proteins when compared with the culture medium of quiescent i.e. presenescent cells and these secreted proteins constitute the so-called senescence-associated secretory phenotype (SASP), also known as the senescence messaging secretome (SMS). SASP components include inflammatory and immune-modulatory cytokines (e.g. IL6 and IL8), growth factors (e.g. IGFBPs), shed cell surface molecules (e.g. TNF receptors) and survival factors. While the SASP exhibits a wide ranging profile, it is not significantly affected by the type of senescence trigger (oncogenic signalling, oxidative stress or DNA damage) or the cell type (epithelial vs. mesenchymal) (Coppe et al. 2008). However, as both oxidative stress and oncogenic signaling induce DNA damage, the persistent DNA damage may be a deciding SASP initiator (Rodier et al. 2009). SASP components function in an autocrine manner, reinforcing the senescent phenotype (Kuilman et al. 2008, Acosta et al. 2008), and in the paracrine manner, where they may promote epithelial-to-mesenchymal transition (EMT) and malignancy in the nearby premalignant or malignant cells (Coppe et al. 2008). Interleukin-1-alpha (IL1A), a minor SASP component whose transcription is stimulated by the AP-1 (FOS:JUN) complex (Bailly et al. 1996), can cause paracrine senescence through IL1 and inflammasome signaling (Acosta et al. 2013).

Here, transcriptional regulatory processes that mediate the SASP are annotated. DNA damage triggers ATM-mediated activation of TP53, resulting in the increased level of CDKN1A (p21). CDKN1A-mediated inhibition of CDK2 prevents phosphorylation and inactivation of the Cdh1:APC/C complex, allowing it to ubiquitinate and target for degradation EHMT1 and EHMT2 histone methyltransferases. As EHMT1 and EHMT2 methylate and silence the promoters of IL6 and IL8 genes, degradation of these methyltransferases relieves the inhibition of IL6 and IL8 transcription (Takahashi et al. 2012). In addition, oncogenic RAS signaling activates the CEBPB (C/EBP-beta) transcription factor (Nakajima et al. 1993, Lee et al. 2010), which binds promoters of IL6 and IL8 genes and stimulates their transcription (Kuilman et al. 2008, Lee et al. 2010). CEBPB also stimulates the transcription of CDKN2B (p15-INK4B), reinforcing the cell cycle arrest (Kuilman et al. 2008). CEBPB transcription factor has three isoforms, due to three alternative translation start sites. The CEBPB-1 isoform (C/EBP-beta-1) seems to be exclusively involved in growth arrest and senescence, while the CEBPB-2 (C/EBP-beta-2) isoform may promote cellular proliferation (Atwood and Sealy 2010 and 2011). IL6 signaling stimulates the transcription of CEBPB (Niehof et al. 2001), creating a positive feedback loop (Kuilman et al. 2009, Lee et al. 2010). NF-kappa-B transcription factor is also activated in senescence (Chien et al. 2011) through IL1 signaling (Jimi et al. 1996, Hartupee et al. 2008, Orjalo et al. 2009). NF-kappa-B binds IL6 and IL8 promoters and cooperates with CEBPB transcription factor in the induction of IL6 and IL8 transcription (Matsusaka et al. 1993, Acosta et al. 2008). Besides IL6 and IL8, their receptors are also upregulated in senescence (Kuilman et al. 2008, Acosta et al. 2008) and IL6 and IL8 may be master regulators of the SASP.

IGFBP7 is also an SASP component that is upregulated in response to oncogenic RAS-RAF-MAPK signaling and oxidative stress, as its transcription is directly stimulated by the AP-1 (JUN:FOS) transcription factor. IGFBP7 negatively regulates RAS-RAF (BRAF)-MAPK signaling and is important for the establishment of senescence in melanocytes (Wajapeyee et al. 2008).

Please refer to Young and Narita 2009 for a recent review.

References

Coppé JP, Patil CK, Rodier F, Sun Y, Muñoz DP, Goldstein J, ... Campisi J (2008). Senescence-associated secretory phenotypes reveal cell-nonautonomous functions of oncogenic RAS and the p53 tumor suppressor. PLoS Biol., 6, 2853-68.

Rodier F, Coppé JP, Patil CK, Hoeijmakers WA, Muñoz DP, Raza SR, ... Campisi J (2009). Persistent DNA damage signalling triggers senescence-associated inflammatory cytokine secretion. Nat. Cell Biol., 11, 973-9.

Kuilman T, Michaloglou C, Vredeveld LC, Douma S, van Doorn R, Desmet CJ, ... Peeper DS (2008). Oncogene-induced senescence relayed by an interleukin-dependent inflammatory network. Cell , 133, 1019-31.

Acosta JC, O'Loghlen A, Banito A, Guijarro MV, Augert A, Raguz S, ... Gil J (2008). Chemokine signaling via the CXCR2 receptor reinforces senescence. Cell, 133, 1006-18.

Takahashi A, Imai Y, Yamakoshi K, Kuninaka S, Ohtani N, Yoshimoto S, ... Hara E (2012). DNA damage signaling triggers degradation of histone methyltransferases through APC/C(Cdh1) in senescent cells. Mol. Cell, 45, 123-31.

Edit history

Date	Action	Author
2012-11-02	Created	Orlic-Milacic M

Date	Action	Author
2013-07-15	Edited	Matthews L, D'Eustachio P
2013-07-15	Authored	Orlic-Milacic M
2013-09-03	Reviewed	Samarajiwa S
2013-09-30	Revised	Orlic-Milacic M
2018-05-24	Modified	Schmidt EE

Elements found in this pathway

Input	UniProt Id	Input	UniProt Id	Input	UniProt Id
CDKN1A	P38936	H2BFS	P57053	HIST1H2BK	O60814
JUN	P05412				

17. Uptake and function of diphtheria toxin (R-HSA-5336415)

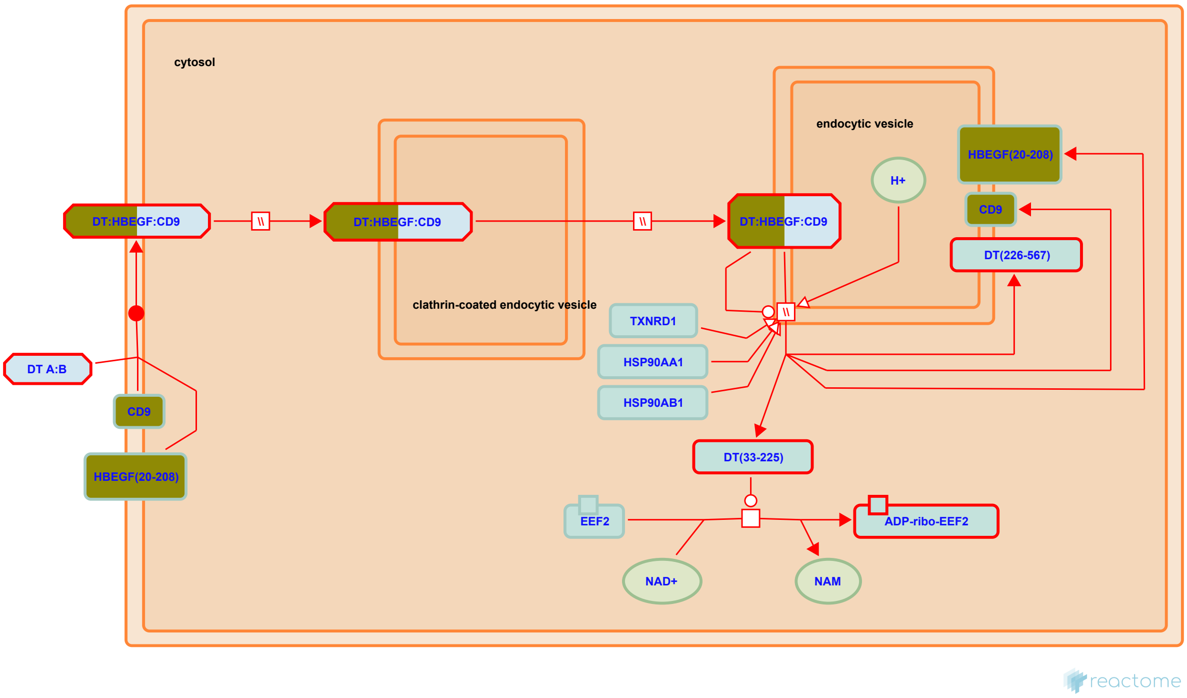

Diseases: diphtheria.

Diphtheria is a serious, often fatal human disease associated with damage to many tissues. Bacteria in infected individuals, however, are typically confined to the lining of the throat or to a skin lesion; systemic effects are due to the secretion of an exotoxin encoded by a lysogenic bacteriophage. The toxin is encoded as a single polypeptide but is cleaved by host furin-like proteases to yield an aminoterminal fragment A and a carboxyterminal fragment B, linked by a disulfide bond. Toxin cleavage can occur when it first contacts the target cell surface, as annotated here, or as late as the point at which fragment A is released into the cytosol. Fragment B mediates toxin uptake into target cell endocytic vesicles, where acidification promotes a conformational change enabling fragment B to form a channel in the vesicle membrane through which fragment A is extruded into the target cell cytosol. Cleavage of the inter-fragment disulfide bond frees DT fragment A, which catalyzes ADP ribosylation of the translation elongation factor 2 (EEF2) in a target cell, thereby blocking protein synthesis. Neither fragment is toxic to human cells by itself (Collier 1975; Pappenheim 1977; Murphy 2011).

References

Collier RJ (1975). Diphtheria toxin: mode of action and structure. Bacteriol Rev, 39, 54-85.

Murphy JR (2011). Mechanism of diphtheria toxin catalytic domain delivery to the eukaryotic cell cytosol and the cellular factors that directly participate in the process. Toxins (Basel), 3, 294-308.

Pappenheimer AM (1977). Diphtheria toxin. Annu. Rev. Biochem., 46, 69-94.

Edit history

Date	Action	Author
2014-03-03	Created	D'Eustachio P
2014-03-05	Edited	D'Eustachio P

Date	Action	Author
2014-03-05	Authored	D'Eustachio P
2014-11-19	Reviewed	Liu S
2015-01-31	Modified	D'Eustachio P

Elements found in this pathway

Input	UniProt Id	Input	UniProt Id	Input	UniProt Id
CD9	P21926	HBEGF	Q99075		

18. MAPK family signaling cascades (R-HSA-5683057)

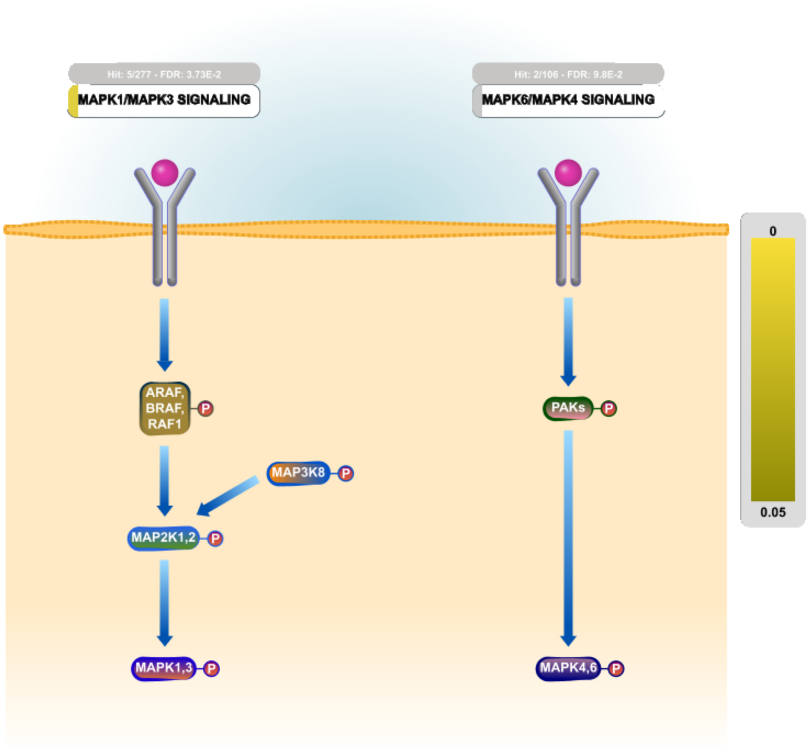

The mitogen activated protein kinases (MAPKs) are a family of conserved protein serine threonine kinases that respond to varied extracellular stimuli to activate intracellular processes including gene expression, metabolism, proliferation, differentiation and apoptosis, among others.

The classic MAPK cascades, including the ERK1/2 pathway, the p38 MAPK pathway, the JNK pathway and the ERK5 pathway are characterized by three tiers of sequentially acting, activating kinases (reviewed in Kryiakis and Avruch, 2012; Cargnello and Roux, 2011). The MAPK kinase kinase kinase (MAPKKK), at the top of the cascade, is phosphorylated on serine and threonine residues in response to external stimuli; this phosphorylation often occurs in the context of an interaction between the MAPKKK protein and a member of the RAS/RHO family of small GTP-binding proteins. Activated MAPKKK proteins in turn phosphorylate the dual-specificity MAPK kinase proteins (MAPKK), which ultimately phosphorylate the MAPK proteins in a conserved Thr-X-Tyr motif in the activation loop.

Less is known about the activation of the atypical families of MAPKs, which include the ERK3/4 signaling cascade, the ERK7 cascade and the NLK cascade. Although the details are not fully worked out, these MAPK proteins don't appear to be phosphorylated downstream of a 3-tiered kinase system as described above (reviewed in Coulombe and Meloche, 2007; Cargnello and Roux, 2011) .

Both conventional and atypical MAPKs are proline-directed serine threonine kinases and, once activated, phosphorylate substrates in the consensus P-X-S/T-P site. Both cytosolic and nuclear targets of MAPK proteins have been identified and upon stimulation, a proportion of the phosphorylated MAPKs relocalize from the cytoplasm to the nucleus. In some cases, nuclear translocation may be accompanied by dimerization, although the relationship between these two events is not fully elaborated (reviewed in Kryiakis and Avruch, 2012; Cargnello and Roux, 2011; Plotnikov et al, 2010).

References

Kyriakis JM & Avruch J (2012). Mammalian MAPK signal transduction pathways activated by stress and inflammation: a 10-year update. Physiol. Rev., 92, 689-737.

Cargnello M & Roux PP (2011). Activation and function of the MAPKs and their substrates, the MAPK-activated protein kinases. Microbiol. Mol. Biol. Rev., 75, 50-83.

Coulombe P & Meloche S (2007). Atypical mitogen-activated protein kinases: structure, regulation and functions. Biochim. Biophys. Acta, 1773, 1376-87.

Plotnikov A, Zehorai E, Procaccia S & Seger R (2011). The MAPK cascades: signaling components, nuclear roles and mechanisms of nuclear translocation. Biochim. Biophys. Acta, 1813, 1619-33.

Edit history

Date	Action	Author
2015-03-10	Authored	Rothfels K
2015-03-11	Created	Rothfels K
2015-04-29	Reviewed	Roskoski R Jr
2018-05-24	Modified	Schmidt EE

Elements found in this pathway

Input	UniProt Id	Input	UniProt Id	Input	UniProt Id
DUSP1	P28562	DUSP4	Q13115	DUSP5	Q16690
DUSP6	Q16828	FOXO3	O43524	HBEGF	Q99075
JUN	P05412				

19. Clearance of Nuclear Envelope Membranes from Chromatin (R-HSA-2993913)

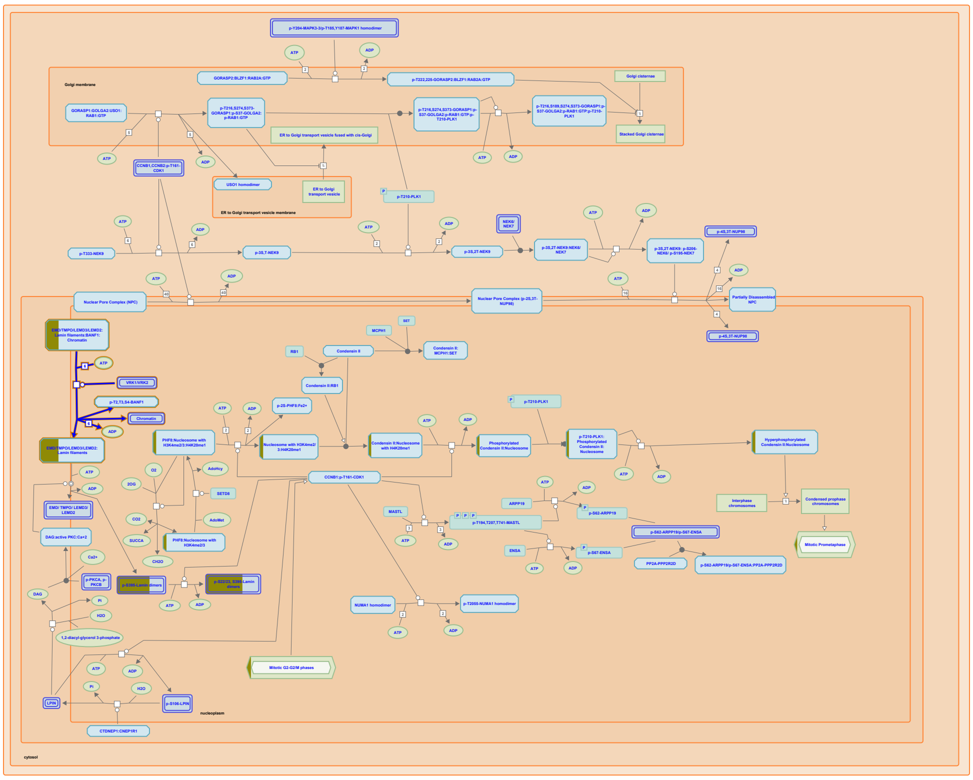

In mitotic prophase, chromatin detaches from the nuclear envelope, and this contributes to the nuclear envelope breakdown. VRK1 (and possibly VRK2) mediated phosphorylation of BANF1 (BAF), a protein that simultaneously interacts with DNA, LEM-domain inner nuclear membrane proteins, and lamins (Zheng et al. 2000, Shumaker et al. 2001, Haraguchi et al. 2001, Mansharamani and Wilson 2005, Brachner et al. 2005) is considered to be one of the key steps in the detachment of the nuclear envelope from chromatin (Bengtsson and Wilson 2006, Nichols et al. 2006, Gorjanacz et al. 2007).

References

Zheng R, Ghirlando R, Lee MS, Mizuuchi K, Krause M & Craigie R (2000). Barrier-to-autointegration factor (BAF) bridges DNA in a discrete, higher-order nucleoprotein complex. Proc. Natl. Acad. Sci. U.S.A., 97, 8997-9002.

Shumaker DK, Lee KK, Tanhehco YC, Craigie R & Wilson KL (2001). LAP2 binds to BAF.DNA complexes: requirement for the LEM domain and modulation by variable regions. EMBO J., 20, 1754-64.

Mansharamani M & Wilson KL (2005). Direct binding of nuclear membrane protein MAN1 to emerin in in vitro and two modes of binding to barrier-to-autointegration factor. J. Biol. Chem., 280, 13863-70.

Haraguchi T, Koujin T, Segura-Totten M, Lee KK, Matsuoka Y, Yoneda Y, ... Hiraoka Y (2001). BAF is required for emerin assembly into the reforming nuclear envelope. J. Cell. Sci., 114, 4575-85.

Dechat T, Gajewski A, Korbei B, Gerlich D, Daigle N, Haraguchi T, ... Foisner R (2004). LAP2alpha and BAF transiently localize to telomeres and specific regions on chromatin during nuclear assembly. J. Cell. Sci., 117, 6117-28.

Edit history

Date	Action	Author
2013-01-22	Created	Orlic-Milacic M
2013-01-23	Edited	Gillespie ME
2013-01-23	Authored	Orlic-Milacic M
2013-01-30	Reviewed	Gorjánácz M, Mattaj IW
2018-05-23	Modified	Schmidt EE

Elements found in this pathway

Input	UniProt Id	Input	UniProt Id	Input	UniProt Id
LMNA	P02545-1, P02545-2				

20. Cell Cycle (R-HSA-1640170)

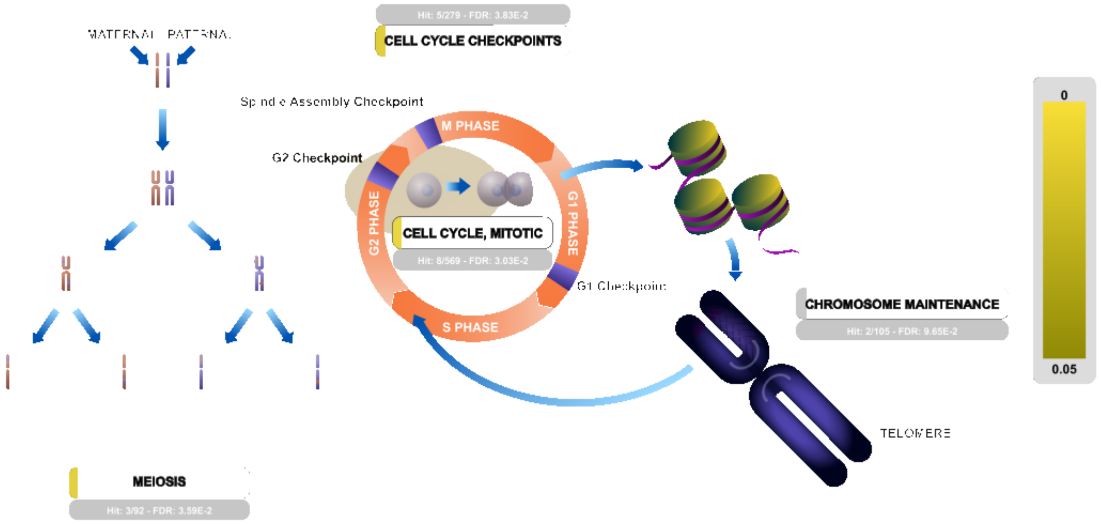

The replication of the genome and the subsequent segregation of chromosomes into daughter cells are controlled by a series of events collectively known as the **cell cycle**. DNA replication is carried out during a discrete temporal period known as the S (synthesis)-phase, and chromosome segregation occurs during a massive reorganization to cellular architecture at mitosis. Two gap-phases separate these major cell cycle events: G1 between mitosis and S-phase, and G2 between S-phase and mitosis. In the development of the human body, cells can exit the cell cycle for a period and enter a quiescent state known as G0, or terminally differentiate into cells that will not divide again, but undergo morphological development to carry out the wide variety of specialized functions of individual tissues.

A family of protein serine/threonine kinases known as the cyclin-dependent kinases (CDKs) controls progression through the cell cycle. As the name suggests, the activity of the catalytic subunit is dependent on binding to a cyclin partner. The human genome encodes several cyclins and several CDKs, with their names largely derived from the order in which they were identified. The oscillation of cyclin abundance is one important mechanism by which these enzymes phosphorylate key substrates to promote events at the relevant time and place. Additional post-translational modifications and interactions with regulatory proteins ensure that CDK activity is precisely regulated, frequently confined to a narrow window of activity.

In addition, genome integrity in the cell cycle is maintained by the action of a number of signal transduction pathways, known as **cell cycle checkpoints**, which monitor the accuracy and completeness of DNA replication during S phase and the orderly chromosomal condensation, pairing and partition into daughter cells during mitosis.

Replication of telomeric DNA at the ends of human chromosomes and packaging of their centromeres into chromatin are two aspects of **chromosome maintenance** that are integral parts of the cell cycle.

Meiosis is the specialized form of cell division that generates haploid gametes from diploid germ cells, associated with recombination (exchange of genetic material between chromosomal homologs).

Edit history

Date	Action	Author
2011-10-10	Edited	Matthews L
2011-10-10	Created	Matthews L
2018-05-24	Modified	Schmidt EE

Elements found in this pathway

Input	UniProt Id	Input	UniProt Id	Input	UniProt Id
CDKN1A	P38936	H2BFS	P57053	HIST1H2BK	O60814
LMNA	P02545-1, P02545-2	PHLDA1	Q8WV24	TUBB2A	Q13885, Q9BVA1

Input	Ensembl Id	Input	Ensembl Id	Input	Ensembl Id
CDKN1A	ENST00000244741, ENSG00000124762				

21. Insulin-like Growth Factor-2 mRNA Binding Proteins (IGF2BPs/IMPs/VICKZs) bind RNA (R-HSA-428359)

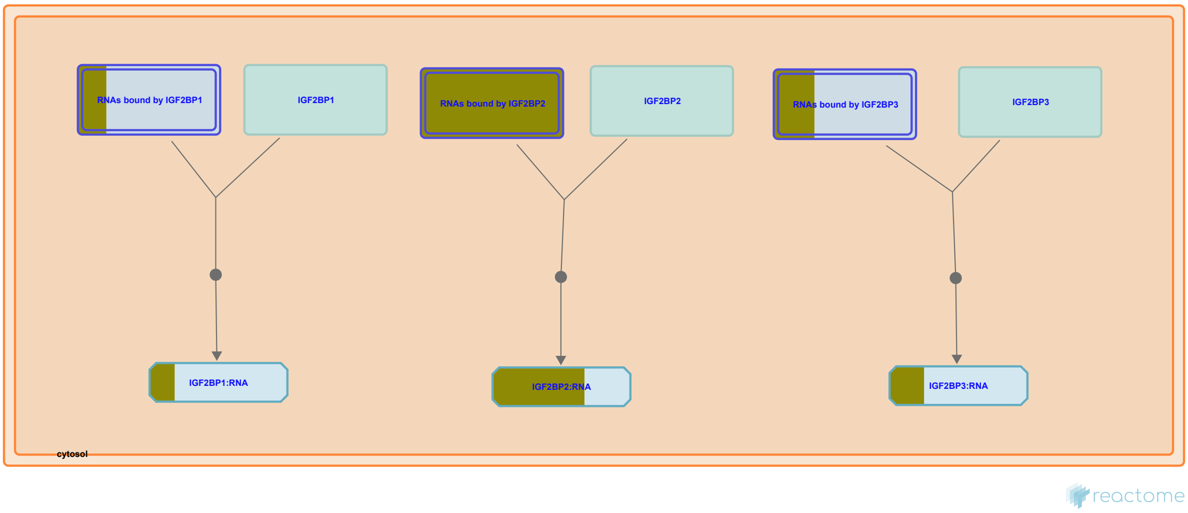

Cellular compartments: cytosol.

Insulin-like Growth Factor-2 mRNA Binding Proteins (IGF2BPs) bind specific sets of RNA and regulate their translation, stability, and subcellular localization. IGF2BP1, IGF2BP2, and IGF2BP3 bind about 8400 protein-coding transcripts. The target RNAs contain the sequence motif CAUH (where H is A, U, or, C) and binding of IGFBPs increases the stability of the target RNAs.

References

Nielsen FC, Nielsen J & Christiansen J (2001). A family of IGF-II mRNA binding proteins (IMP) involved in RNA trafficking. Scand J Clin Lab Invest Suppl, 234, 93-9.

Nielsen J, Christiansen J, Lykke-Andersen J, Johnsen AH, Wewer UM & Nielsen FC (1999). A family of insulin-like growth factor II mRNA-binding proteins represses translation in late development. Mol Cell Biol, 19, 1262-70.

Vikesaa J, Hansen TV, Jønson L, Borup R, Wewer UM, Christiansen J & Nielsen FC (2006). RNA-binding IMPs promote cell adhesion and invadopodia formation. EMBO J, 25, 1456-68.

Oberman F, Rand K, Maizels Y, Rubinstein AM & Yisraeli JK (2007). VICKZ proteins mediate cell migration via their RNA binding activity. RNA, 13, 1558-69.

Yisraeli JK (2005). VICKZ proteins: a multi-talented family of regulatory RNA-binding proteins. Biol Cell, 97, 87-96.

Edit history

Date	Action	Author
2009-07-05	Edited	May B
2009-07-05	Authored	May B
2009-07-05	Created	May B
2010-05-30	Reviewed	Chao JA, Singer RH
2016-12-29	Modified	D'Eustachio P

Elements found in this pathway

Input	Ensembl Id	Input	Ensembl Id	Input	Ensembl Id
IGF2	ENST00000337883, ENST00000381406				

22. ERKs are inactivated (R-HSA-202670)

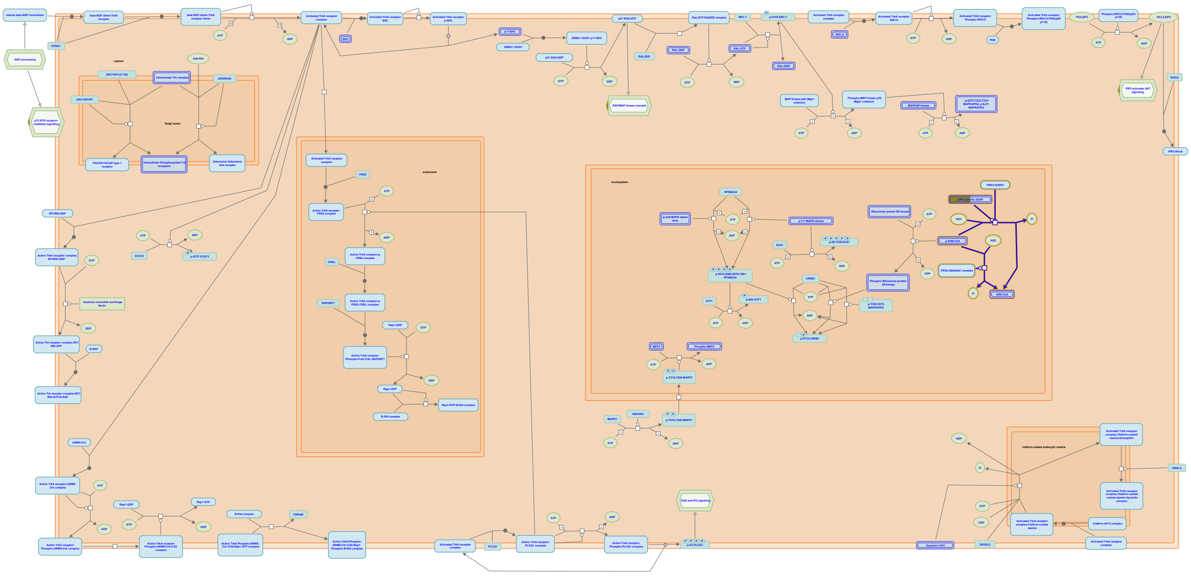

MAP Kinases are inactivated by a family of protein named MAP Kinase Phosphatases (MKPs). They act through dephosphorylation of threonine and/or tyrosine residues within the signature sequence -pTXpY- located in the activation loop of MAP kinases (pT=phosphothreonine and pY=phosphotyrosine). MKPs are divided into three major categories depending on their preference for dephosphorylating; tyrosine, serine/threonine and both the tyrosine and threonine (dual specificity phoshatases or DUSPs). The tyrosine-specific MKPs include PTP-SL, STEP and HePTP, serine-/threonine-specific MKPs are PP2A and PP2C, and many DUSPs acting on MAPKs are known. Activated MAP kinases trigger activation of transcription of MKP genes. Therefore, MKPs provide a negative feedback regulatory mechanism on MAPK signaling, by inactivating MAPKs via dephosphorylation, in the cytoplasm and the nucleus. Some MKPs are more specific for ERKs, others for JNK or p38MAPK.

References

Farooq A & Zhou MM (2004). Structure and regulation of MAPK phosphatases. Cell Signal, 16, 769-79.

Amit I, Citri A, Shay T, Lu Y, Katz M, Zhang F, ... Yarden Y (2007). A module of negative feedback regulators defines growth factor signaling. Nat Genet, 39, 503-12.

Edit history

Date	Action	Author
2007-11-08	Reviewed	Greene LA
2007-11-08	Created	Jassal B
2018-06-04	Modified	Croft D

Elements found in this pathway

Input	UniProt Id	Input	UniProt Id	Input	UniProt Id
DUSP4	Q13115	DUSP6	Q16828		

23. Activation of HOX genes during differentiation (R-HSA-5619507)

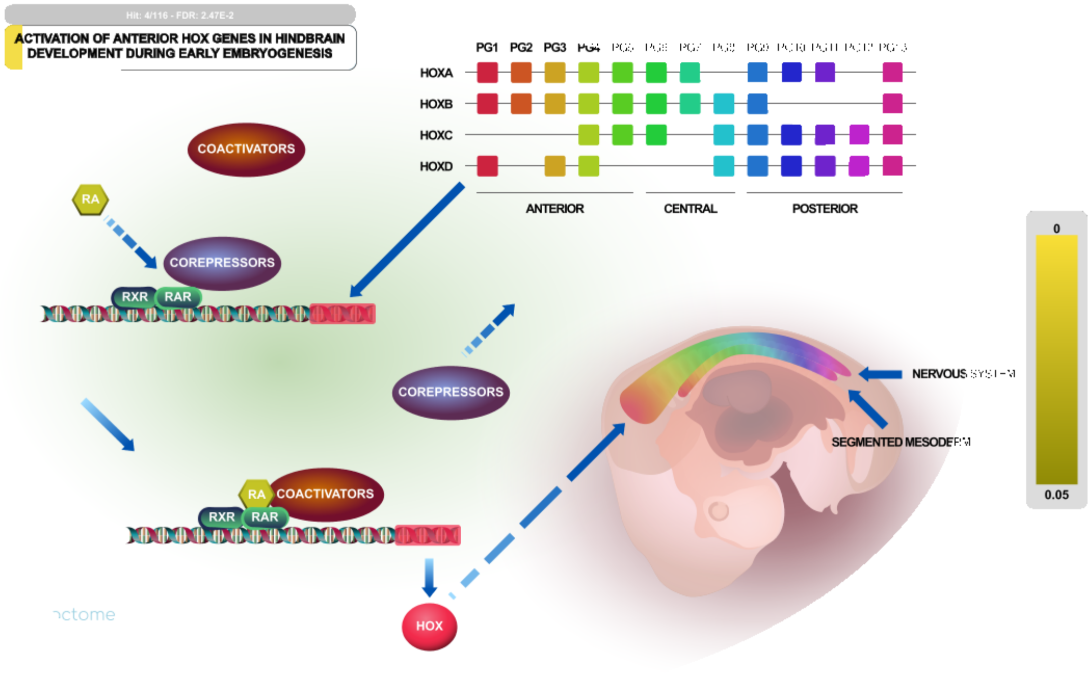

Hox genes encode proteins that contain the DNA-binding homeobox motif and control early patterning of segments in the embryo as well as later events in development (reviewed in Rezsohazy et al. 2015). Mammals have 39 Hox genes arrayed in 4 linear clusters, with each cluster containing 9 to 11 genes. Based on homologies, the genes have been assigned to 13 paralogous groups. The nomenclature of Hox genes uses a letter to indicate the cluster and a number to indicate the paralog group. For example, HOXA4 is the gene in cluster A that is most similar with genes of paralog group 4 from other clusters.

One of the most striking aspects of mammalian Hox gene function is the mechanism of their activation during embryogenesis: the order of genes in a cluster correlates with the timing and location of their activation such that genes at the 3' end of a cluster are activated first and genes at the 5' end of a cluster are activated last. (5' and 3' refer to the transcriptional orientation of the genes in the cluster.) Because development of segments of the embryo proceeds from anterior to posterior this means that the anterior boundaries of expression of 3' genes are more anterior (rostral) and the anterior boundaries of expression of 5' genes are more posterior (caudal).
Expression of HOX genes initiates in the posterior primitive streak at the beginning of gastrulation at approximately E7.5 in mouse. As gastrulation proceeds, further 5' genes are sequentially activated and they too undergo the same chromatin changes and migration. After formation of the axis of the embryo, similar waves of activation of HOXA and HOXD clusters occur in developing limbs beginning at about E9. Retinoids, especially all trans retinoic acid (atRA), participate in initiating the process via retinoid receptors. Other factors such as FGFs and Wnt, also regulate Hox expression. After activation, Hox genes participate in maintaining their own expression (autoregulation), activating later, 5' Hox genes, and repressing prior, 3' Hox genes (crossregulation). Differentiation of embryonal carcinoma cells and embryonic stem cells in response to retinoic acid is used to model the process in vitro (reviewed in Gudas et al. 2013).

Activation of Hox genes is accompanied by a change from bivalent chromatin to euchromatin (reviewed in Soshnikova and Duboule 2009). Bivalent chromatin has extensive methylation of lysine-9 on histone H3 (H3K9me3), a repressive mark, with interspersed punctate regions of methylation of lysine-4 on histone H3 (H3K4me2, H3K4me3), an activating mark. Euchromatization initiates at the 3' ends of clusters and proceeds towards the 5' ends, with the euchromatin migrating to an active region of the nucleus (reviewed in Montavon and Duboule 2013). This change in chromatin reflects a loss of H3K27me3 and a gain of H3K4me2,3. Polycomb repressive complexes bind H3K27me3 and are responsible for maintenance of repression, KDM6A and KDM6B histone demethylases remove H3K27me3, and members of the trithorax family of histone methylases (KMT2A, KMT2C, KMT2D) methylate H3K4.

References

Soshnikova N & Duboule D (2009). Epigenetic regulation of vertebrate Hox genes: a dynamic equilibrium. Epigenetics, 4, 537-40.

Andrey G & Duboule D (2014). SnapShot: Hox gene regulation. Cell, 156, 856-856.e1.

Montavon T & Duboule D (2013). Chromatin organization and global regulation of Hox gene clusters. Philos. Trans. R. Soc. Lond., B, Biol. Sci., 368, 20120367.

Mallo M, Wellik DM & Deschamps J (2010). Hox genes and regional patterning of the vertebrate body plan. Dev. Biol., 344, 7-15.

Rezsohazy R, Saurin AJ, Maurel-Zaffran C & Graba Y (2015). Cellular and molecular insights into Hox protein action. Development, 142, 1212-1227.

Edit history

Date	Action	Author
2014-08-22	Edited	May B
2014-08-22	Authored	May B
2014-08-24	Created	May B
2015-05-13	Reviewed	Rezsohazy R, Blasi F
2017-03-13	Modified	May B

Elements found in this pathway

Input	UniProt Id	Input	UniProt Id	Input	UniProt Id
EGR2	P11161	H2BFS	P57053	HIST1H2BK	O60814
JUN	P05412				

24. Activation of anterior HOX genes in hindbrain development during early embryogenesis (R-HSA-5617472)

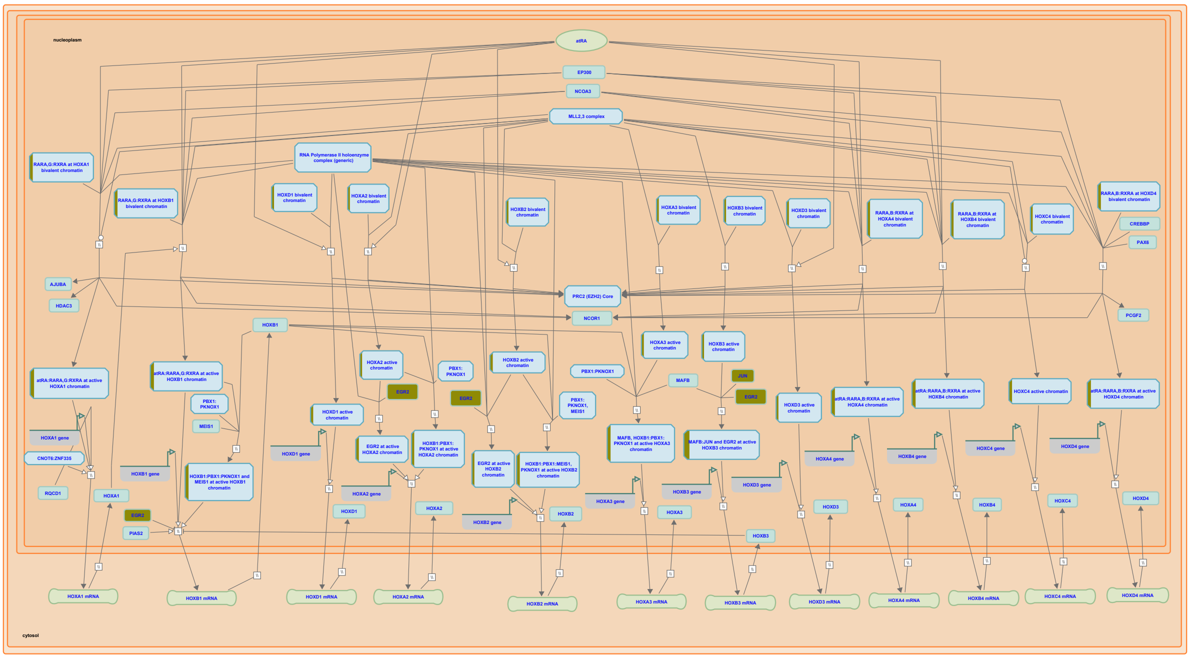

In mammals, anterior Hox genes may be defined as paralog groups 1 to 4 (Natale et al. 2011), which are involved in development of the hindbrain through sequential expression in the rhombomeres, transient segments of the neural tube that form during development of the hindbrain (reviewed in Alexander et al. 2009, Soshnikova and Duboule 2009, Tumpel et al. 2009, Mallo et al. 2010, Andrey and Duboule 2014). Hox gene activation during mammalian development has been most thoroughly studied in mouse embryos and the results have been extended to human development by in vitro experiments with human embryonal carcinoma cells and human embryonic stem cells.

Expression of a typical anterior Hox gene has an anterior boundary located at the junction between two rhombomeres and continues caudally to regulate segmentation and segmental fate in ectoderm, mesoderm, and endoderm. Anterior boundaries of expression of successive Hox paralog groups are generally separated from each other by 2 rhombomeres. For example, HOXB2 is expressed in rhombomere 3 (r3) and caudally while HOXB3 is expressed in r5 and caudally. Exceptions exist, however, as HOXA1, HOXA2, and HOXB1 do not follow the rule and HOXD1 and HOXC4 are not expressed in rhombomeres. Hox genes within a Hox cluster are expressed colinearly: the gene at the 3' end of the cluster is expressed earliest, and hence most anteriorly, then genes 5' are activated sequentially in the same order as they occur in the cluster.

Activation of expression occurs epigenetically by loss of polycomb repressive complexes and change of bivalent chromatin to active chromatin through, in part, the actions of trithorax family proteins (reviewed in Soshnikova and Duboule 2009). Hox gene expression initiates in the posterior primitive streak that will contribute to extraembryonic mesoderm. Expression then extends anteriorly into the cells that will become the embryo, where expression is first observed in presumptive lateral plate mesoderm and is transmitted to both paraxial mesoderm and neurectoderm formed by gastrulation along the primitive streak (reviewed in Deschamps et al. 1999, Casaca et al. 2014).

Prior to establishment of the rhombomeres, expression of HOXA1 and HOXB1 is initiated near the future site of r3 and caudally by a gradient of retinoic acid (RA). (Mechanisms of retinoic acid signaling are reviewed in Cunningham and Duester 2015.) The RA is generated by the ALDH1A2 (RALDH2) enzyme located in somites flanking the caudal hindbrain and degraded by CYP26 enzymes expressed initially in anterior neural ectoderm of the early gastrula and then throughout most of the hindbrain (reviewed in White and Schilling 2008). HOXA1 with PBX1,2 and MEIS2 directly activate transcription of ALDH1A2 to maintain retinoic acid synthesis in the somitic mesoderm (Vitobello et al. 2011). Differentiation of embryonal carcinoma cells and embryonic stem cells in response to retinoic acid is used to model the process of differentiation in vitro (reviewed in Soprano et al. 2007, Gudas et al. 2013).

HOXA1 appears to set the anterior limit of HOXB1 expression (Barrow et al. 2000). HOXB1 initiates expression of EGR2 (KROX20) in presumptive r3. EGR2 then activates HOXA2 expression in r3 and r5 while HOXB1, together with PBX1 and MEIS:PKNOX1 (MEIS:PREP), activates expression of HOXA2 in r4 and caudal rhombomeres. AP-2 transcription factors maintain expression of HOXA2 in neural crest cells (Maconochie et al. 1999). HOXB1 also activates expression of HOXB2 in r3 and caudal rhombomeres. EGR2 negatively regulates HOXB1 so that by the time rhombomeres appear, HOXB1 is restricted to r4 and HOXA1 is no longer detectable (Barrow et al. 2000). EGR2 and MAFB (Kreisler) then activate HOXA3 and HOXB3 in r5 and caudal rhombomeres. Retinoic acid activates HOXA4, HOXB4, and HOXD4 in r7, the final rhombomere. HOX proteins, in turn, activate expression of genes in combination with other factors, notably members of the TALE family of transcription factors (PBX, PREP, and MEIS, reviewed in Schulte and Frank 2014, Rezsohazy et al. 2015). HOX proteins also participate in non-transcriptional interactions (reviewed in Rezsohazy 2014). In zebrafish, Xenopus, and chicken factors such as Meis3, Fgf3, Fgf8, and vHNF regulate anterior hox genes (reviewed in Schulte and Frank 2014), however less is known about the roles of homologous factors in mammals.

Mutations in HOXA1 in humans have been observed to cause developmental abnormalities located mostly in the head and neck region (Tischfield et al. 2005, Bosley et al. 2008). A missense mutation in HOXA2 causes microtia, hearing impairment, and partially cleft palate (Alasti et al. 2008). A missense mutation in HOXB1 causes a similar phenotype to the Hoxb1 null mutation in mice: bilateral facial palsy, hearing loss, and strabismus (improper alignment of the eyes) (Webb et al. 2012).

References

Tümpel S, Wiedemann LM & Krumlauf R (2009). Hox genes and segmentation of the vertebrate hindbrain. Curr. Top. Dev. Biol., 88, 103-37.

Alexander T, Nolte C & Krumlauf R (2009). Hox genes and segmentation of the hindbrain and axial skeleton. Annu. Rev. Cell Dev. Biol., 25, 431-56.

Soshnikova N & Duboule D (2009). Epigenetic regulation of vertebrate Hox genes: a dynamic equilibrium. Epigenetics, 4, 537-40.

Andrey G & Duboule D (2014). SnapShot: Hox gene regulation. Cell, 156, 856-856.e1.

Mallo M, Wellik DM & Deschamps J (2010). Hox genes and regional patterning of the vertebrate body plan. Dev. Biol., 344, 7-15.

Edit history

Date	Action	Author
2014-08-08	Edited	May B
2014-08-08	Authored	May B
2014-08-09	Created	May B
2015-05-13	Reviewed	Blasi F
2015-11-15	Authored	Rezsohazy R
2016-07-30	Modified	Gillespie ME

Elements found in this pathway

Input	UniProt Id	Input	UniProt Id	Input	UniProt Id
EGR2	P11161	H2BFS	P57053	HIST1H2BK	O60814
JUN	P05412				

25. Nuclear Envelope Reassembly (R-HSA-2995410)

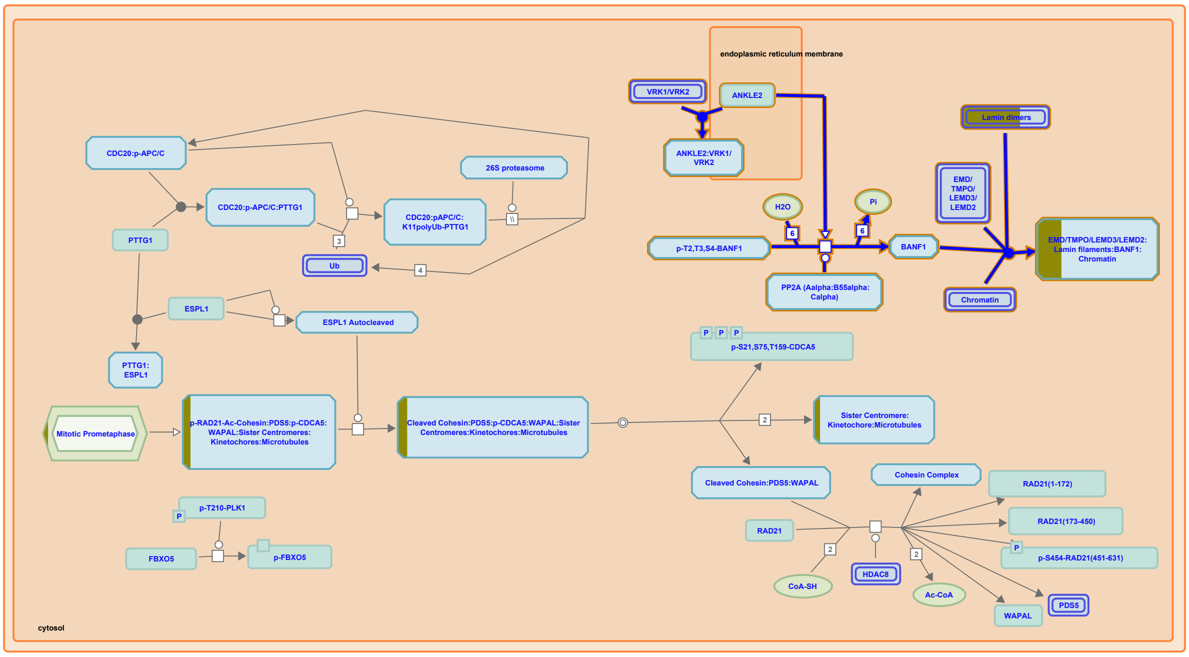

The reassembly of the nuclear envelope (NE) around separated sister chromatids begins in late anaphase and is completed in telophase. Nuclear pore complexes (NPCs) reassemble and insert into the reforming nuclear envelope (reviewed by Guttinger et al. 2009).

References

Güttinger S, Laurell E & Kutay U (2009). Orchestrating nuclear envelope disassembly and reassembly during mitosis. Nat. Rev. Mol. Cell Biol., 10, 178-91.

Edit history

Date	Action	Author
2013-01-23	Edited	Gillespie ME
2013-01-23	Authored	Orlic-Milacic M
2013-01-23	Created	Orlic-Milacic M
2013-01-30	Reviewed	Gorjánácz M, Mattaj IW
2018-05-24	Modified	Schmidt EE

Elements found in this pathway

Input	UniProt Id	Input	UniProt Id	Input	UniProt Id
LMNA	P02545-1, P02545-2				

6. Identifiers found

Input	UniProt Id	Input	UniProt Id	Input	UniProt Id
CCL3	P10147	CD9	P21926	CDKN1A	P38936
DUSP1	P28562	DUSP4	Q13115	DUSP5	Q16690
DUSP6	Q16828	EGR1	P18146	EGR2	P11161
FOXO3	O43524	H2BFS	P57053	HBEGF	Q99075
HIST1H2BK	O60814	HMOX1	P09601	ISG20	Q96AZ6
JUN	P05412	LMNA	P02545-1	MCL1	Q07820
MT2A	P02795	PHLDA1	Q8WV24	PMAIP1	Q13794
SQSTM1	Q13501	TUBB2A	Q13885, Q9BVA1		

Input	Ensembl Id	Input	Ensembl Id	Input	Ensembl Id
CCL3	ENSG00000277632	CDKN1A	ENSG00000124762	EGR1	ENSG00000120738
FOXO3	ENSG00000118689	HMOX1	ENSG00000100292	IGF2	ENST00000337883, ENST00000381406
ISG20	ENSG00000172183	MCL1	ENSG00000143384	MT2A	ENSG00000125148
PMAIP1	ENSG00000141682				

7. Identifiers not found

EGR3	FHL1	FOSL1	GADD45B	IER2	MIR22HG	PHLDA2	SCGB2A2
