# Supplementary material for: Mebendazole is unique among tubulin-active drugs in activating the MEK–ERK pathway
Source: Sci Rep. 2020 Aug 4;10:13124. doi: 10.1038/s41598-020-68986-0 (PMC7403428; doi:10.1038/s41598-020-68986-0)
Supplement: Supplementary file 2 — Supplementary Figures. [file 41598_2020_68986_MOESM2_ESM.pdf]

Supplementary Figure S1a: Effect of MBZ and a panel of TBAs on tubulin polymerisation.

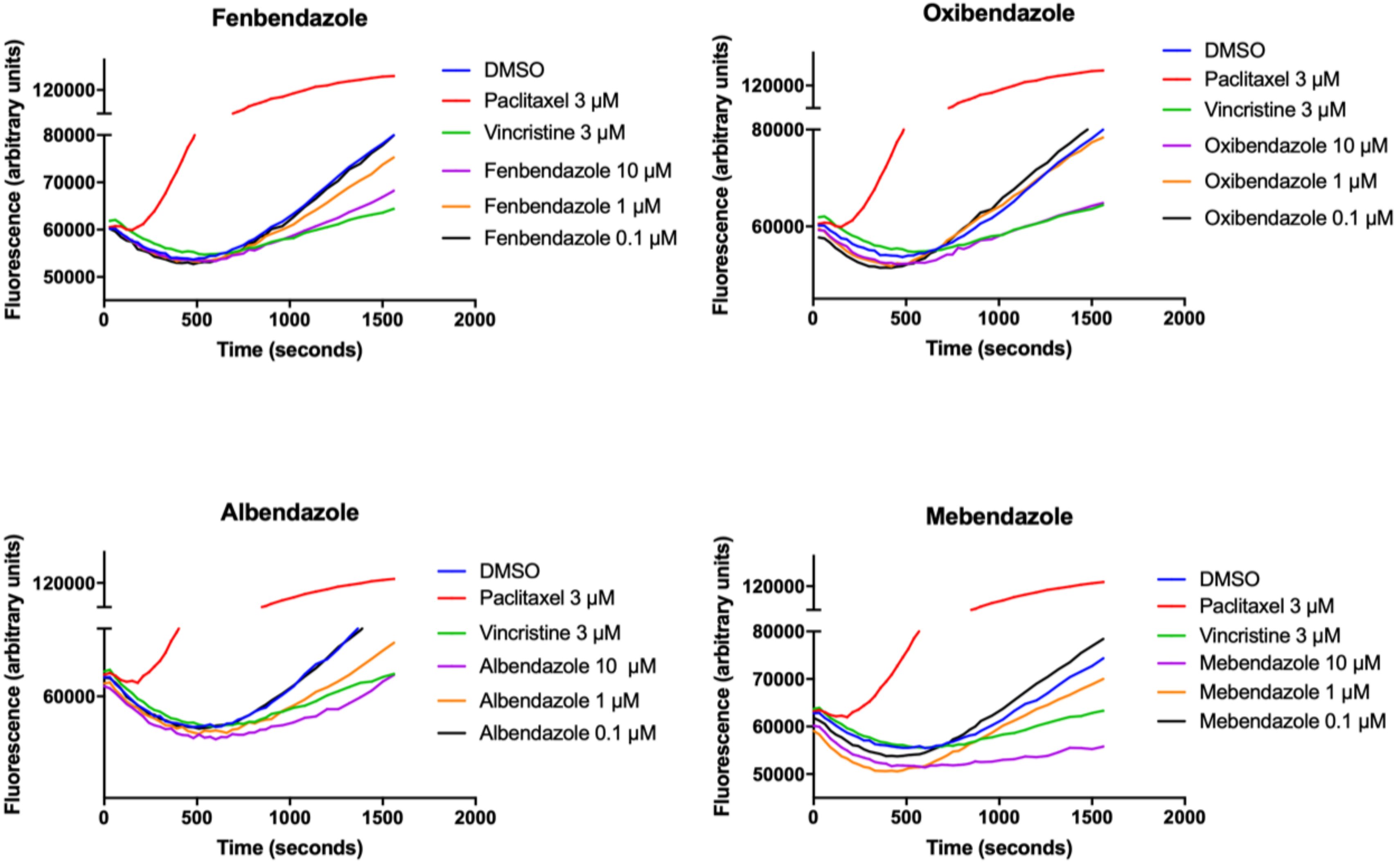

Curves above DMSO control indicate inhibition of tubulin depolymerisation, curves below inhibition of tubulin polymerisation.

Supplementary Figure S1b : Effect of MBZ and a panel of TBAs on tubulin polymerisation.

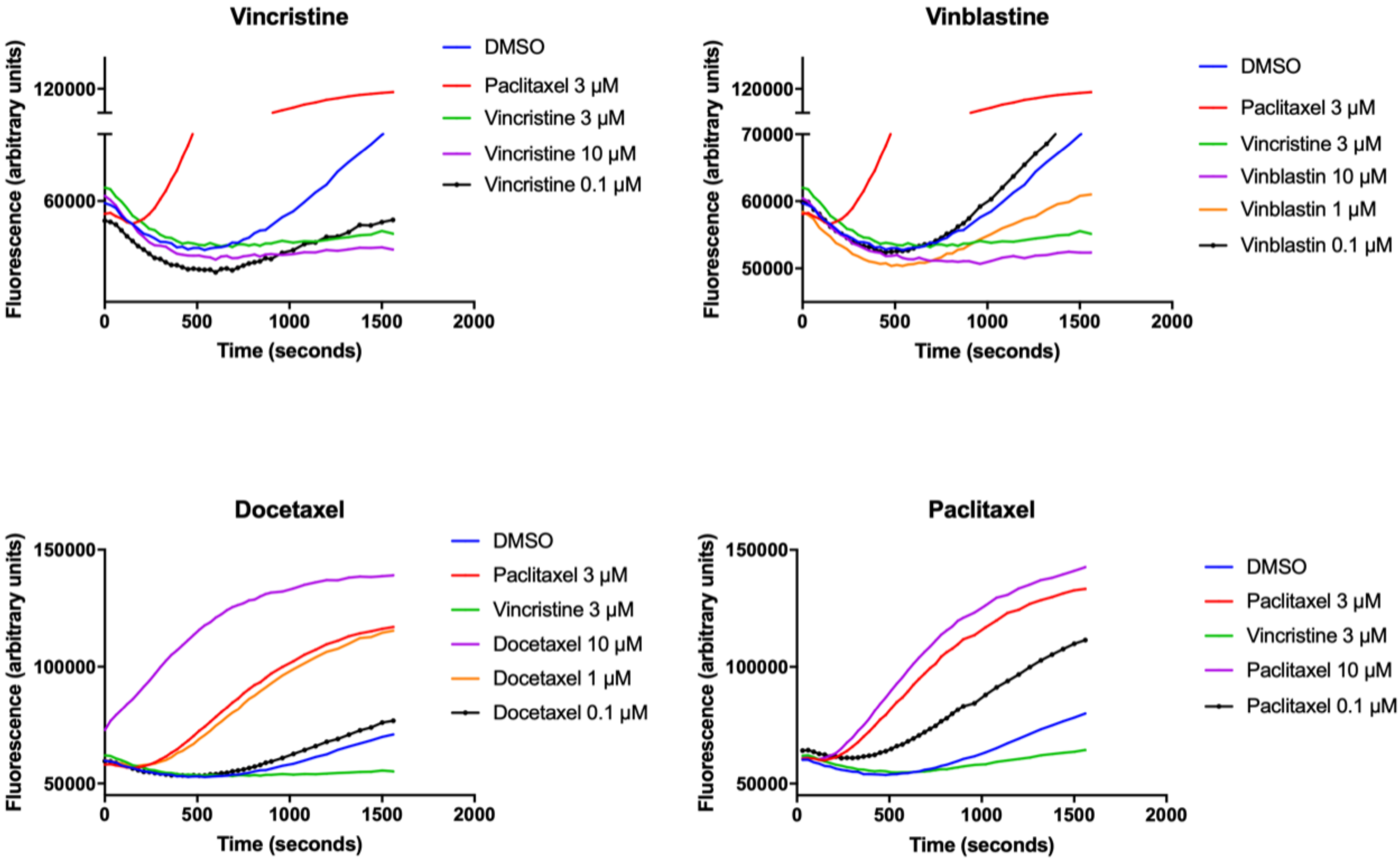

Curves above DMSO control indicate inhibition of tubulin depolymerisation, curves below inhibition of tubulin polymerisation.

Supplementary Figure S2:
Effect of MBZ and a panel of TBAs on cell cycle distribution.

Cell cycle arrest (shift in phase distribution) relative to untreated and DMSO control is observed for the TBAs at the assayed concentrations.

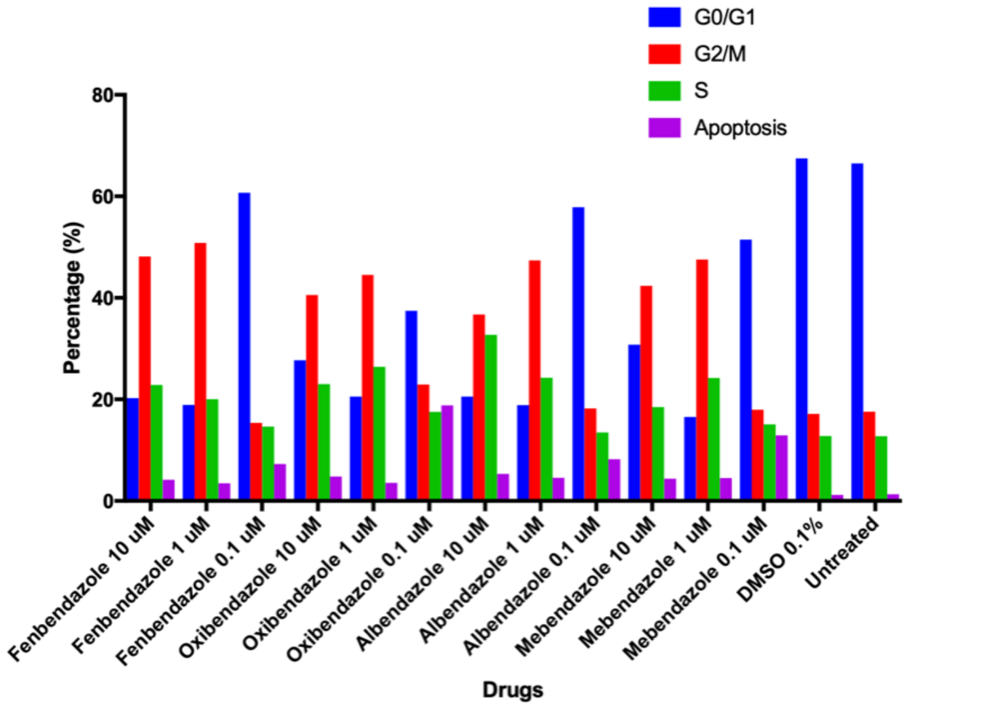

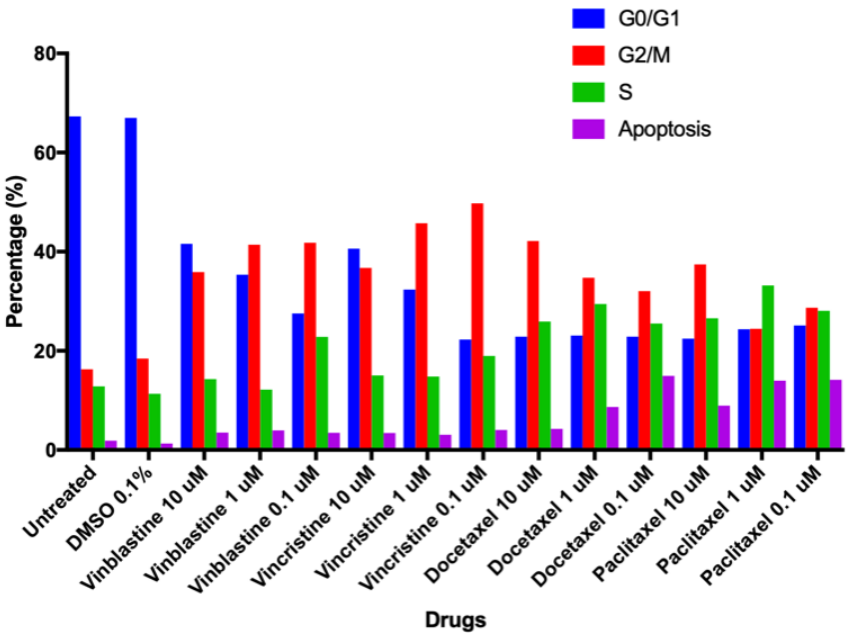

Supplementary Figure S3: Effect of MBZ and LPS/IFN gamma on MAPK phosphoprotein activity in naive THP-1 monocytes over time.

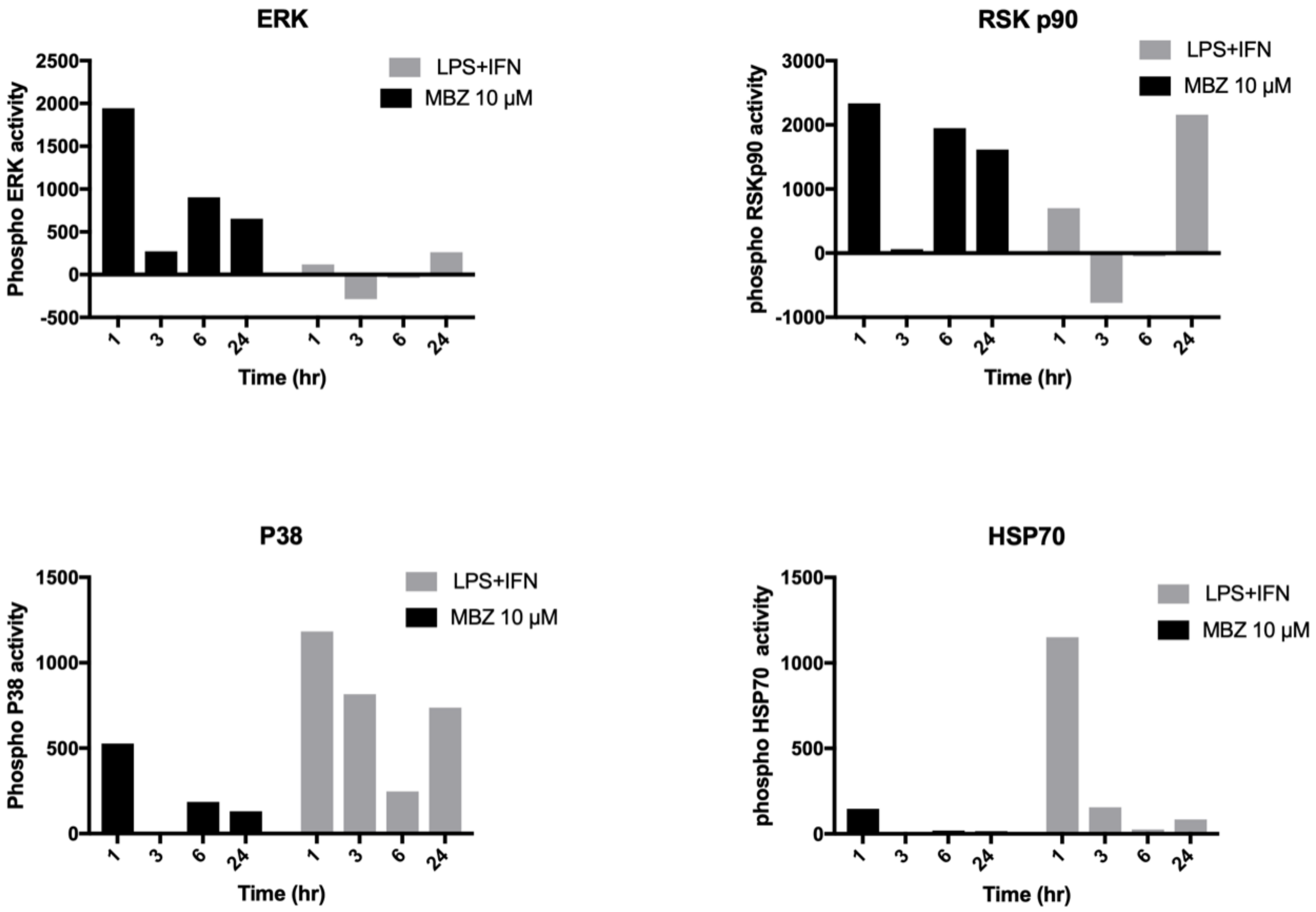

Supplementary Figure S4: Hypothetical mechanism for MBZ induced effects in autoimmune disease.

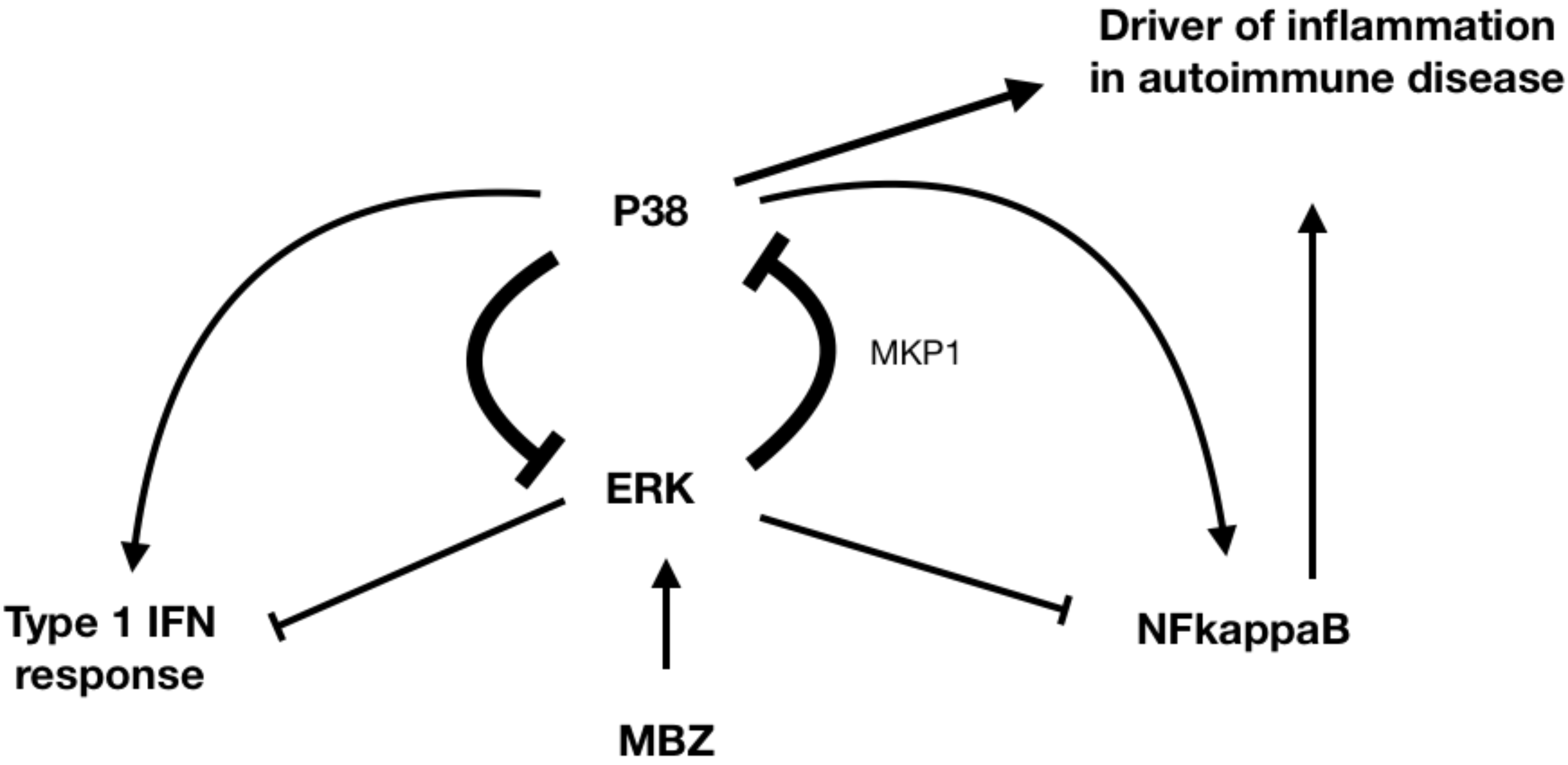

ERK can exert negative feedback on p38/JNK driven inflammation through stabilization of MKP1. The resulting decrease in p38 and JNK down-regulates the pro-inflammatory response and type 1 IFN response. In addition, ERK has been shown to inhibit NFKB.
